# Vessel strike encounter risk model informs mortality risk for endangered North Atlantic right whales along the United States east coast

**DOI:** 10.1038/s41598-024-84886-z

**Published:** 2025-01-03

**Authors:** Hannah Blondin, Lance P. Garrison, Jeffrey D. Adams, Jason J. Roberts, Caroline P. Good, Meghan P. Gahm, Niki E. Lisi, Eric M. Patterson

**Affiliations:** 1 University of Miami University of Miami Cooperative Institute for Marine & Atmospheric Studies (CIMAS), Miami, FL 33149 USA; 2 NOAA Fisheries, Southeast Fisheries Science Center, Miami, FL 33149 USA; 3 Marine Geospatial Ecology Laboratory, Duke University, Durham, NC 27708 USA; 4 Office of Protected Resources, NOAA Fisheries, Silver Spring, MD 20910 USA; 5 Southeast Fisheries Science Center, Marine Mammal and Turtle Division, National Marine Fisheries Service, 75 Virginia Beach Dr, Miami, FL 33149 USA

**Keywords:** North Atlantic right whales, Risk model, Human wildlife conflict, Vessel strike, Endangered species, Conservation, Marine biology, Ocean sciences

## Abstract

Vessel strikes are a critical threat to endangered North Atlantic right whales (*Eubalaena glacialis*), significantly contributing to their elevated mortality. Accurate estimates of these mortality rates are essential for developing effective management strategies to aid in the species’ recovery. This study enhances existing vessel strike models by incorporating detailed regional data on vessel traffic characteristics as well as whale distribution and behavior. Our model assesses the spatial and temporal variability in vessel strike risk along the U.S. east coast apportioned into three vessel length classes (26–65 feet, 65–350 feet, > 350 feet). By including regional right whale depth distributions and parameterizing potential whale avoidance based on factors such as descent rate, bottom depth, and vessel speed and size, the model provides a refined estimation of mortality risk. We also address the underrepresentation of smaller vessel activity via a correction factor, offering a more accurate annual mortality risk estimate for each vessel size class. These findings highlight that vessels > 350 feet in length pose the greatest risk to right whales. Simulations of reduced vessel speeds indicate that speed measures can mitigate mortality rates; however, residual risk remains even at speeds of 10 knots or less suggesting limitations to this mitigation approach.

**Supplementary Information:**

The online version contains supplementary material available at 10.1038/s41598-024-84886-z.

## Introduction

As the oceans become more industrialized, marine megafauna become increasingly vulnerable to adverse human-wildlife interactions^1–3^. One such interaction is a vessel strike, or the collision between an animal and a vessel. Vessel strikes present a particular risk to species that spend much of their time at the ocean surface, such as air-breathing animals (e.g., whales and turtles^1,4,5^) and those that feed, rest, and/or thermoregulate at shallow depths^5^. Vessel strikes often result in lethal or sub-lethal injuries, due to blunt force trauma and/or laceration injuries and can have significant impacts on the survival, reproduction, and recovery of vulnerable populations^6^. As a result, vessel strikes can have disproportionate effects on already small populations, such as the North Atlantic right whale (*Eubalaena glacialis;*hereafter ‘right whale’), where even a single human-caused mortality puts additional strain on the endangered population^7,8^. Right whales are listed as endangered under the U.S. Endangered Species Act^9^and are considered critically endangered according to the International Union for Conservation of Nature (IUCN)^10^, with an estimated 372 individuals remaining (95% CI: from 360 to 383 individuals)^8^. An observed average of 2.4 right whales per year suffered vessel strike mortalities or serious injuries from 2016 to 2020^7^; however, this is a minimum estimate. Many vessel strike-related deaths and serious injuries likely go unreported; best estimates indicate that only 36% of all right whale mortalities are observed^11^.

Significant uncertainty remains regarding the factors that increase vessel collision risk for large whales. This complicates our ability to understand when and where strikes are likely to occur, the impact on affected populations, and how best to mitigate these impacts. For large whales in particular, behavior can vary greatly in both horizontal and vertical space^12^. Horizontal movement and behavior, including long-distance migrations, are influenced by a range of biotic and abiotic factors such as food availability, reproductive needs, and ocean currents and temperature^13–15^. Vertical use of the water column also varies and can depend on geographic location, behavioral state (e.g., foraging, breeding), and the location of prey in the water column^16,17^. Human use of the oceans is also difficult to observe and predict. United States Coast Guard (USCG) regulations require most vessels greater than or equal to 65 feet in length to carry automatic identification system (AIS) devices (33 CFR § 164.46), but most vessels under 65 feet are not required to carry AIS and are therefore underrepresented in these data^18^. Spatial and temporal variation in vessel traffic patterns, while easier to observe due to AIS coverage, aren’t always predictable and can depend on weather, sea conditions, economic factors, and port activities^19–21^.

When assessing vessel strike risk to large whales, it is important to consider known areas with significant uncertainty. For example, we have a limited understanding of both how whales perceive and react to vessel traffic and the factors that may influence a whales’ ability to successfully avoid a collision. Additionally, given that a strike event does occur, whether or not a whale suffers a mortality or is severely injured depends on a number of factors including vessel speed, vessel size, and the species of whale involved in the interaction^22–24^. These interactions often occur offshore and because whale carcasses often sink and/or are scavenged and surveys are limited, many collisions and resulting mortalities are not documented and are therefore under-reported^11,25,26^. Furthermore, vessel strikes aren’t always apparent from external observation and require necropsy to accurately assess injuries and determine if a vessel strike occurred^26^. For example, if a whale suffers a blunt force trauma injury there may be no visible, external evidence of harm, unlike laceration injuries from contact with the vessel hull, propeller, rudder or skeg which leave externally visible injuries^27^. These factors make it difficult to estimate the actual number of vessel strikes a given population suffers each year, yet vessel strikes are likely to contribute to the rapid decline of this already endangered species.

To address these uncertainties and provide information for management and conservation efforts, vessel strike risk models have been used to assess and predict the likelihood of collisions between vessels and marine megafauna. These models quantify the potential for vessel strikes to occur in specific geographic areas and during specific times of year^19,28–31^. Vessel strike risk models have become increasingly probabilistic, in order to incorporate the many uncertainties listed above, while producing estimates of mortality for various populations (e.g^30,32^). Key components of vessel strike risk models include (1) habitat and distribution data on the species of interest; often a species distribution model that incorporates biological data, environmental data, and spatial location data on the species, (2) species specific factors such as time spent at surface-depths, swim speed, and behavior by geographic region, season, or time of day, (3) vessel traffic data such as transit distance, location, speed, and vessel draft (i.e., the depth of the vessel below the waterline) and (4) collision probability, or the likelihood that a collision occurs between an individual vessel and animal. However, vessel strike risk models to date have used a simple scalar to estimate rates of whale avoidance of vessels (e.g., 55% in^28,32^) that does not account for species-specific behavior such as diving descent rates, nor the non-linear relationship between encounter rate and avoidance that is suggested by the literature. Existing model formulations also do not accurately account for heterogeneity in risk from different vessel sizes and their speeds, nor do they correct for the fact that vessel traffic from smaller vessels (26–65 feet) are underrepresented in AIS data. Finally, current vessel strike risk models rely on an estimated relationship between vessel speed and strike outcome that is based on data from over a decade ago, despite the fact that additional data exists to better inform our understanding of this aspect of the factors that contribute to vessel strike mortality^24^.

In this study, we make significant improvements to existing and widely applied vessel strike encounter risk models by incorporating an improved method for accounting for whale avoidance of vessels based on empirical data (McKenna et al.^47^) as well as an improved understanding of vessel strike lethality as a function of vessel speed, vessel size, and whale species^24^. Finally, to ensure our results are meaningful in terms of overall absolute vessel strike risk, here, we correct for the underrepresentation of traffic from smaller vessels (25–65 feet) in AIS data. We use this newly refined vessel strike encounter risk model to assess the risk of lethal right whale vessel strikes along the U.S. East Coast. Vessel strikes have a significant impact on the health and recovery of right whales, and is one of the two main causes (in addition to entanglement in fishing gear) of the ongoing unusual mortality event, which includes lethal and sublethal injuries^33–36^. We employ updated data on right whale density, distribution^37^and behavior, the frequency, density, and characteristics of vessel traffic based on vessel length, and lethality probabilities^24^. We aim to identify areas and times of greatest risk by vessel size class and explore how reductions in vessel speed may reduce annual mortality rates.

## Methods

### Encounter risk model

**Fig. 1:**
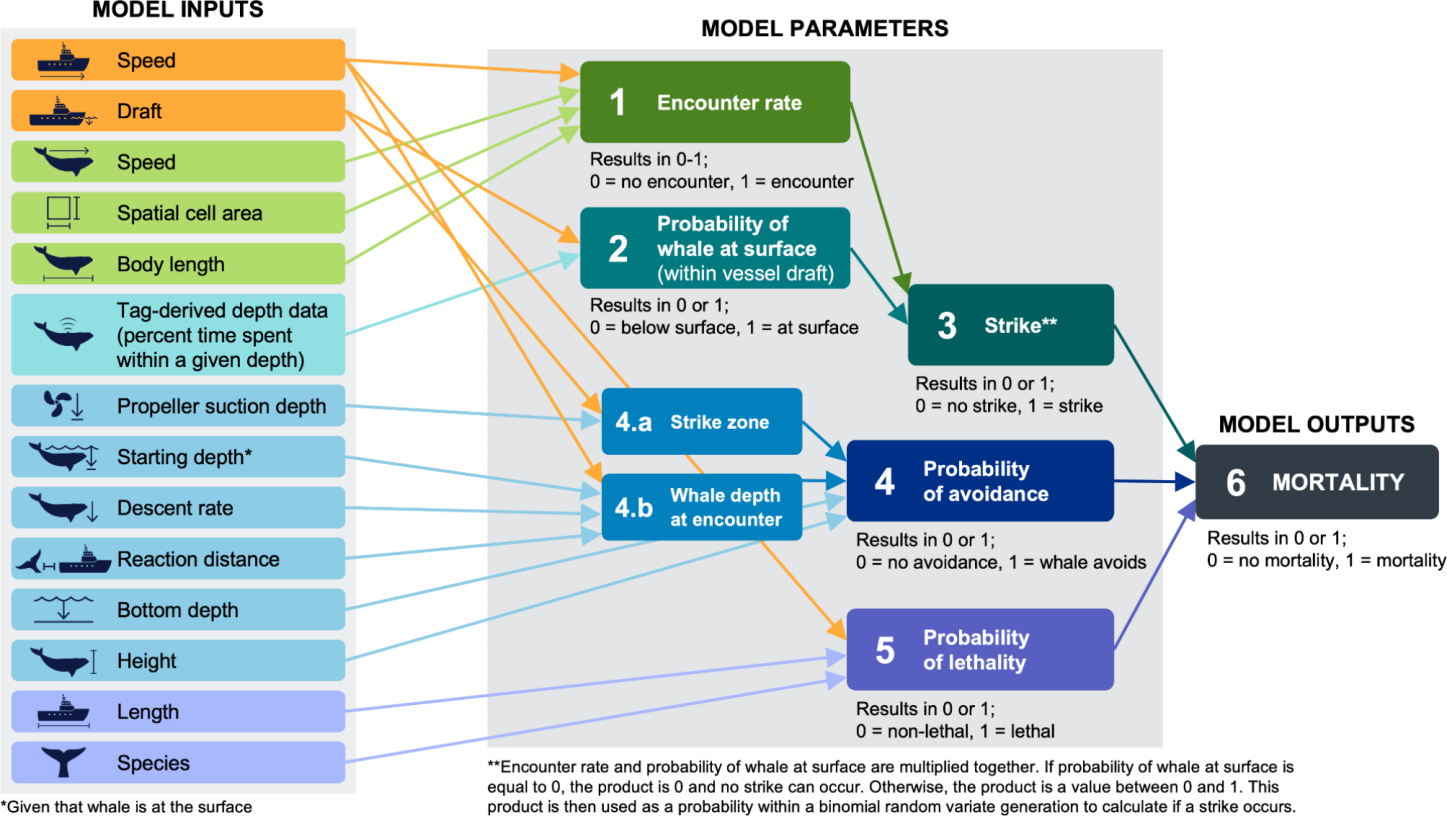
Conceptual diagram outlining the key components of the vessel strike encounter risk model. Model inputs, model parameters, and model outputs are grouped together. Colors are representative of specific model parameters (e.g., model inputs that are in green boxes are used to calculate the encounter rate). Vessel speed and vessel draft are in orange because they are used in the calculation of multiple parameters. Mortality only equals 1 when a strike is equal to 1, the probability of avoidance is equal to 0, and the probability of lethality is equal to 1.

The updated encounter risk model builds upon methods described in Garrison et al.^30^, and uses the same primary equation and model parameters (Eq. 1, Fig. 1), where total mortality is calculated each year for each of three vessel size classes. The model is based on several inputs including vessel and right whale data, characteristics, and behavior (Fig. 1). Mortality is calculated on a transit-by-transit basis where model inputs are synthesized and result in a binary output of mortality, given the presence of a whale in the same defined area (i.e., spatial cell) as the transit.

The model is structured as an iterative simulation model, with probabilistic parameters drawn at each iteration as random variates from appropriate sampling distributions (Eq. 1, Fig. 1). We ran 1000 iterations (i.e., bootstraps) to account for differences in these randomly selected parameters (further details provided in the following sections). The encounter risk model functions as described in Eq. 1:

1 $$\:{M}_{year}=\:{\sum\:}_{i=1}^{12}{\sum\:}_{j=1}^{n}{\sum\:}_{k=1}^{n}{{\lambda}{{{e}^{t}}}_{ijk}}\:\cdot\:\:{{p}_{strike\:depth}}_{ijk}\cdot\:{\left(1-{p}_{avoid}\right)}_{ijk}\cdot\:{{p}_{lethality}}_{ijk}\cdot\:{N_{w_{ij}}}$$

where mortality (i.e., number of mortalities within a given year, $\:{M}_{year}$) is the sum of the total number of mortalities within each track ($\:k$), within each spatial cell ($\:j$) and within each month ($\:i$), and is a function of per-capita encounter rate ($\:{\lambda\:}_{e}t$), the probability that a whale will successfully avoid the strike($\:{p}_{avoid}$), the probability that a whale is located within the draft of the vessel ($\:{p}_{strike\:depth}$), the probability of mortality given a strike ($\:{{p}_{lethality}}_{ijk}$ ), and the number of whales ($\:{N}_{w}$) in a 10 × 10 km spatial grid cell. We also calculate the mean of $\:{M}_{year}$, where cumulative mortality rate per year is averaged across years. The parameters in Eq. 1 are explained in the next sections.

#### Encounter rate

Encounter rate ($\:{\lambda\:}_{e}t$) is based on a two-dimensional model^19,29,30^ where rate of encounter between an individual whale and an individual vessel assumes that both the whale and the vessel are moving randomly in a defined spatial area, in this case the individual 10 × 10 km spatial grid cell. Encounter rate is calculated for a time,$\:\:t$, which is equivalent to the number of seconds it takes for the vessel to transit the grid cell. The encounter rate is equal to one when the vessel is within one body length (13.5 m) of the whale (i.e., irrespective of whale avoidance, and the probability that the whale is located at a depth within the draft of a vessel at this point in the model). We then calculate the other parameters of the model such as the probability of whale avoidance and the probability that a whale is at the surface, as detailed below, to infer that a strike has occurred. Additional information on the two-dimensional encounter risk model can be found in Garrison et al.^30^, Martin et al.^19^, and Crum et al.^29^.

#### Right whale density model

To inform the vessel strike risk model with respect to the presence of right whales, we use the most recent right whale density model (version 12) produced by Roberts et al.^37^, which was built using right whale survey data spanning 1999–2020. Over the last decade, right whales have been increasingly documented in Mid-Atlantic U.S. waters^38^, as well as within newly identified foraging habitat south of Nantucket Island in Southern New England^36^. Therefore, to account for these recent changes in distribution, we based the vessel strike encounter risk model on the “2010–2019” summary era version of the right whale density model (spanning October 2010 - September 2020) produced by Roberts et al.^37^. The full model spans from southern Florida to the Nova Scotian Shelf. Separate models were fit for primary habitat regions in: (1) the Northeast U.S. (hereafter, “Northeast”) covering Georges Bank and the Gulf of Maine and ranging from North of Nantucket shoals to the Nova Scotian Shelf, (2) the Mid-Atlantic U.S. (hereafter, “Mid-Atlantic”) and southern New England covering the area from Cape Hatteras, NC to Nantucket Shoals, and (3) the Southeast U.S. (hereafter, “Southeast”) covering the area from Florida to Cape Hatteras, NC. Predictions in all regions are made on a 5 × 5 km grid. Here, we aggregated predictions for the number of whales ($\:{N}_{w}$) on a 10 × 10 km grid to match the resolution of previous analyses (e.g., 28; Fig. 1).

#### Vessel data

**Fig. 2:**
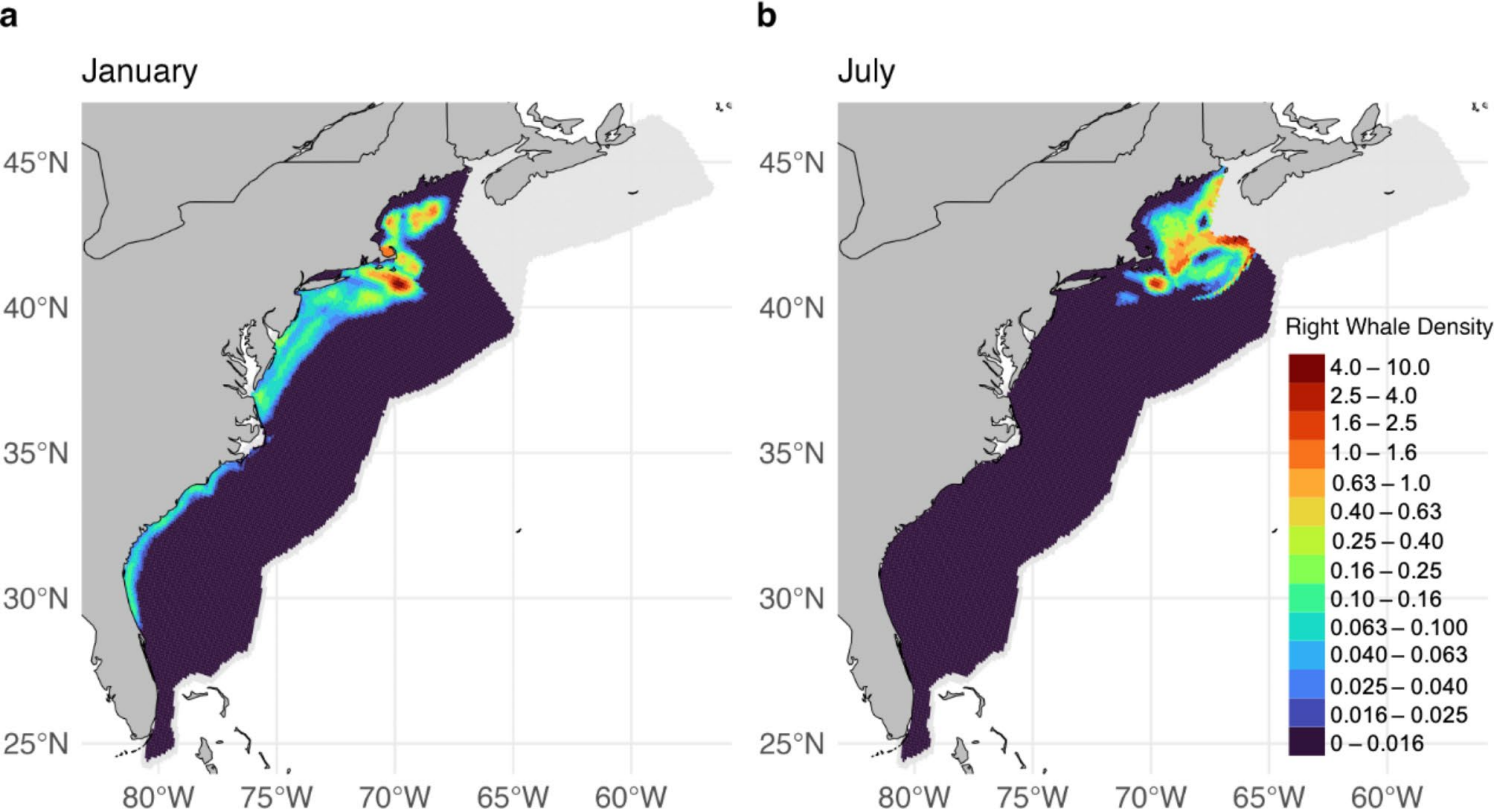
Right whale spatial density in two example months: (**a**) January and (**b**) July (Roberts et al^37^.). This figure was generated in R version 4.2.3 (https://www.r-project.org/). Country-level boundaries were sourced from Massicotte P, South A (2024). rnaturalearth: World Map Data from Natural Earth. R package version 1.0.

Vessel data used for the model followed the same methods as Garrison et al.^30^. Automatic Identification System (AIS) data collected from low orbiting satellite constellations (ORBCOMM) and terrestrial stations^39^were used in the model to characterize vessel traffic. Data included calendar years 2017–2019 and 2021–2022. As a result of decreases in maritime activities during COVID-19 in 2020, which were apparent in our data from visual inspection and have been reported elsewhere^40,41^, we excluded 2020 from all analyses. Data on vessel characteristics such as vessel type and vessel length were collated via the AIS data and from a third-party registration vessel registration database^42^. We further processed individual vessel tracks by removing erroneous speeds (i.e., > 50 knots and < 0.2 knots) and removing tracks with elapsed times of 0 s. We also imputed missing vessel characteristics such as vessel length, beam, and draft by summarizing these characteristics within vessel categories (e.g., bulk containers, towing/pushing, passenger (cruise)) and assigning vessels with unknown characteristics from random draws of vessels in the same categories with known characteristics. Vessel tracks were then spatially intersected with and summarized by the same 10 × 10 km grid as the right whale density model (Fig. 2). More information can be found in Garrison et al.^30^.

To examine differences in vessel strike mortality based on vessel size, we divided vessels into three general size classes based on vessel length: (1) Small/Medium vessels: vessels 26 to 65 feet in length (2) Large vessels: vessels 65 to 350 feet in length, and (3) Ocean-going vessels (OGVs): vessels over 350 in length. We used these vessel length bins to match a previous analysis from Garrison et al.^24^, which found a significant relationship between vessel length as categorical variable, vessel speed, and whale mortality (more details provided in Sect. 2.1.6). Most vessels under 65 feet are not required to carry AIS data (33 CFR 164.46), and therefore are largely under-represented in the AIS data. To adjust for this, we derived a correction factor based on 2022 vessel registration data and applied it to the AIS data for vessels under 65 feet. We used these registration data to estimate the proportion of unique vessels within each vessel size class-category combination (e.g., passenger vessels 26–65 feet, fishing vessels 65–350 feet) compared to the AIS data. These registration data included: Atlantic Highly Migratory Species (HMS) Permits (HMS angling, HMS charter/headboat, general (Atlantic tunas and/or swordfish), harpoon (Atlantic tunas and/or swordfish), and trap); NMFS Southeast Regional Office (SERO) Federal Permits for fishing in the Exclusive Economic Zone (EEZ), and NOAA Fisheries Greater Atlantic Region (GARFO) vessel permits for federally managed species three to 200 nautical miles offshore^39^. This ratio was used for all study years as a correction factor and applied to scale the number of interactions for individual whales within the encounter risk model to more accurately assess the impact of the Small/Medium size class on mortality due to vessel strike. Within this correction factor, we assume that AIS data for Small/Medium-sized vessels are spatially representative of vessels within this size class for the spatial distribution of the unaccounted-for vessels.

#### Probability of a whale at strike depth

**Fig. 3:**
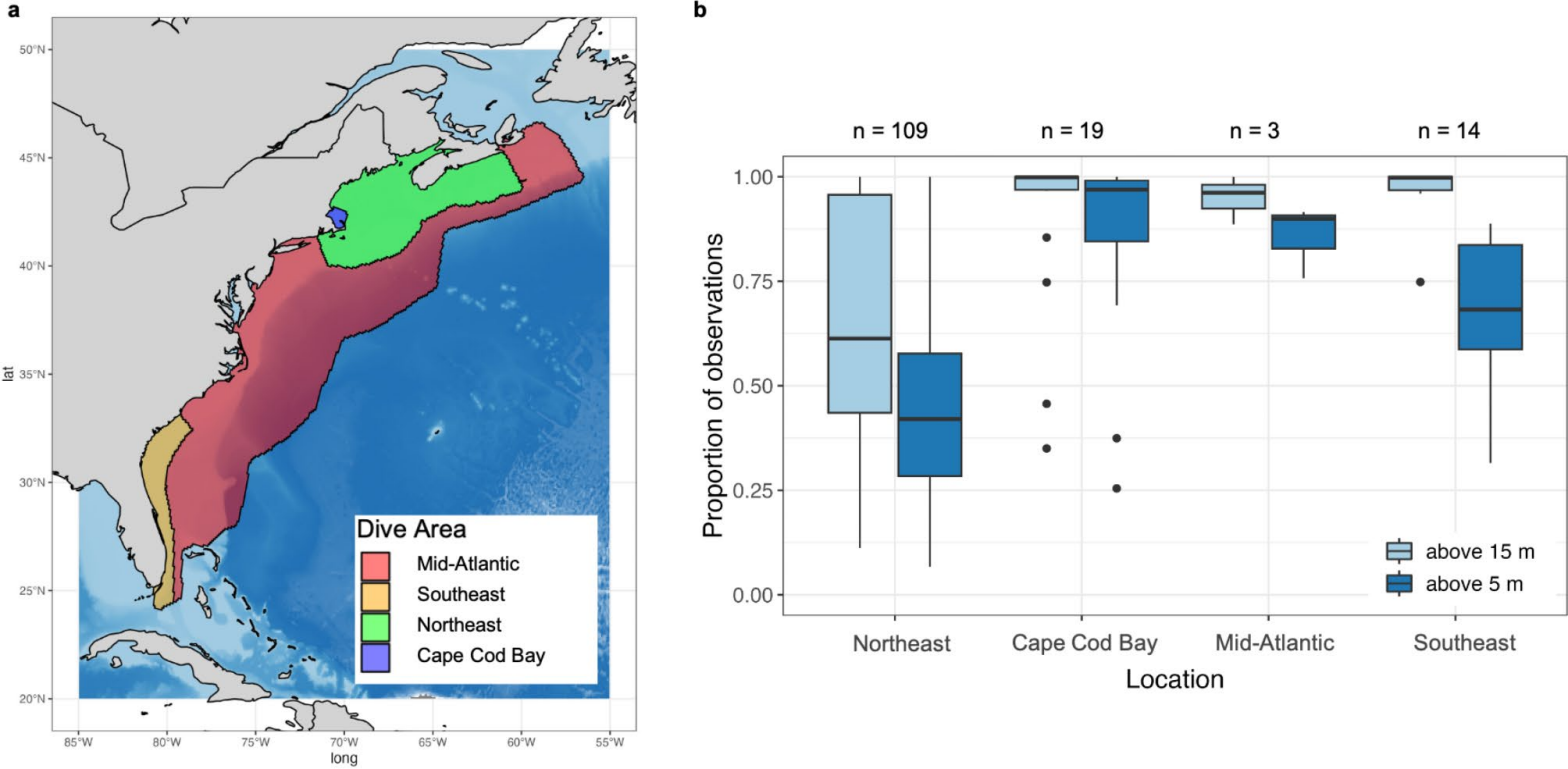
(**a**) Map of study area showing regional sub-areas and primary behavior within each sub-area. (**b**) Boxplots showing the proportion of time spent by tagged individual right whales at depths of 15 m or less and 5 m or less by geographic region. This figure was generated in R version 4.2.3 (https://www.r-project.org/). Country-level boundaries were sourced from Massicotte P, South A (2024). rnaturalearth: World Map Data from Natural Earth. R package version 1.0.

The probability that a whale depth is within the draft of a vessel ($\:{\rho\:}_{strike\:depth}$) is based on empirical data from 146 depth-recording tags^17,43–46^. Tag data were aggregated by geographic location: Northeast, Cape Cod Bay, Mid-Atlantic, and Southeast (Fig. 3a, Table S1, Table S2). These regions represent areas where right whales generally exhibit different behaviors and life histories (Fig. 3a, Table S1)^37,38^. The Southeast region is characterized by reproductive behavior and comprises the population’s calving grounds. The Northeast and Cape Cod Bay regions are both characterized by foraging behavior, but the particular foraging technique used differs between the regions given differences in depth and the availability of preferred prey, *Calanus* copepods. The Mid-Atlantic region is generally characterized by migratory behaviors, where whales travel between the calving grounds in the Southeast and the foraging grounds in the Northeast. For each region, the weighted proportion of time spent above 5 and 15 m was calculated to reflect time within the draft of smaller and larger vessels respectively. Proportions were weighted by the duration of each individual tag (Table S2). In the encounter risk model, the probability of a whale occurring at strike depth randomly draws from a binomial distribution, with probabilities set to the proportion of time above vessel drafts from the tag data. Encounter rate and probability of a whale at surface are then multiplied together. If the probability of a whale at the surface is equal to 0, the product is 0 and no strike can occur. Otherwise, the product is a value between 0 and 1. This product is then used as a probability within a binomial random variate generation to calculate if a strike occurs (Fig. 1).

#### Probability of avoidance

The probability of avoidance function here was adapted from McKenna et al.^47^, where the authors documented dive responses of blue whales to oncoming commercial shipping vessels in Southern California. These authors observed vertical avoidance in blue whales 55% of the time, but did not find any evidence of lateral avoidance in response to oncoming vessels. Here we apply the same equation Mckenna et al.^47^ derived from their empirical work, but include data relevant and specific to right whales, such as whale height, whale depth, and whale descent rate.

We calculate *P*_*avoid*_ as a binary variable as follows:

2 $$\:{P\left(avoid\right)}_{jk}=\left\{\begin{array}{c}1\\\:0\end{array}\right.\:\:{Whale\:Depth}_{k}>{Strike\:Zone}_{k}\:\&\:{Bottom\:Depth}_{j}>{Strike\:Zone}_{k}+Whale\:Height$$

where if $\:WhaleDepth$ is greater than the $\:StrikeZone$ of a given vessel and $\:WhaleHeight$ and $\:StrikeZone$ + $\:WhaleHeight$ is less than total $\:BottomDepth$ of spatial cell $\:j$, the whale is considered to have successfully avoided a vessel strike, and the probability of avoidance ($\:{\rho\:}_{avoid}$) equals 1. If $\:WhaleDepth$ is less than $\:StrikeZone$ + $\:WhaleHeight$ or if $\:StrikeZone$ + $\:WhaleHeight$ is greater than $\:BottomDepth$ of spatial cell $\:\:j$ the whale is considered to have been struck by the vessel as it lacks sufficient vertical space to escape, and the probability of avoidance ($\:{\rho\:}_{avoid}$) equals 0. $\:WhaleHeight$is defined by the average height of an adult right whale, or 3 m^48^. $\:BottomDepth$ for a given cell $\:j$is defined as the mean depth at 15 arc-second resolution^49^. All cells with a mean depth of less than 3 m were removed from the analysis as it would not be possible for a right whale to swim there.

$\:WhaleDepth$ is defined by Eq. 3 as follows:

3 $$\:{Whale\:Depth}_{k}=\:{Start\:Depth}_{k}+\left({Descent\:Rate}_{k}\:\times\:\:\frac{{Reaction\:Distance}_{k}}{{Vessel\:Speed}_{k}}\right)\:$$

where $\:WhaleDepth$ is the depth of a whale from the surface in the water column, given a randomly-selected start depth $\:StartDepth$, $\:DescentRate$, and $\:ReactionDistance$), as well as the $\:VesselSpeed$ within the transit $\:k$ of a vessel in a given cell $\:j$. The random start depth ranges from 0 to the maximum draft of the vessel (5 m for vessels under 350 feet and 15 m for vessels over 350 feet). Whale descent rates used in our model range from 0.81 to 2.0 m/s and were based on observed descent rates of right whales^45^. Whale reaction distances ranged 10 to 1200 m and vessel speeds were based on actual vessel transects from AIS data (more details below). Probability of avoidance is only relevant to the equation, when the encounter rate and the probability of a whale at the surface determine that the whale is within one body length of the vessel, the whale is within the draft of a vessel, and a strike occurs.

The $\:StrikeZone$ parameter is defined in Eq. 4 as follows:

4 $$\:{Strike\:Zone}_{k}={Vessel\:Draft}_{k}\:\times\:\:Propeller\:Suction\:Depth$$

where $\:StrikeZone$is defined as vessel draft multiplied by a scalar adjustment for propeller suction depth. For Small/Medium and Large vessels, the scaler is equal to 1, meaning the strike zone is simply equivalent to the size class vessel draft. However, for OGVs, the scaler is equal to 2, meaning that the strike zone is equal to twice the draft due to hydrodynamics of those vessels^50^, or 30 m.

#### Probability of lethality

The probability of lethality, given a vessel strike occurs, was modeled in Garrison et al.^24^ and applied here. Garrison et al.^24^, as shown in Eq. 5, used a logistic regression and found a significant relationship between the probability of whale mortality given that a strike occurs and vessel size (as a categorical variable), vessel speed, and whale species (i.e., whether the species involved in the interaction is a humpback whale or is another large whale species, see^30^ for more details) and the interaction between vessel speed and whale species. When applied here, vessel size and speed for each calculation are taken directly from the AIS data of the vessel segment. Probability of avoidance is only relevant to the equation, when the encounter rate is equal to 1 (i.e., the whale is within one body length of the vessel), the probability of the whale at the surface is equal to 1 (i.e., the whale is at the surface within the draft of the vessel at the time of the encounter), and the probability of avoidance is equal to 0 (i.e., the whale does not avoid the oncoming vessel).

5 $$\:{P\left(lethality\right)}_{k}=\left\{\begin{array}{c}1\\\:0\end{array}\right.\:\:{Fate\:\sim\:Vessel\:Speed}_{k}+{Vessel\:Size}_{k}+Whale\:Species+{Vessel\:Speed}_{k}\times\:Whale\:Species$$

### Speed simulation analysis

We also simulated the effect of reduced vessel speeds on mortality rates on annual rates of mortality to determine the maximum benefit achieved from speed reductions. First, tracks from vessels within specified spatial areas traveling over 10 knots were identified. Next a speed between 9.5 and 10.0 knots was randomly selected and applied to the track. The model was then run a second time, with all randomly-selected parameters besides speed (e.g., whale depth, whale descent rate, etc.) held equal. Certain AIS-equipped vessels transiting over 10-knots were excluded from the speed simulation because they are exempt from North Atlantic right whale vessel speed regulations (50 CFR 224.105). Speed restrictions do not apply to U.S. military vessels, vessels owned, operated, or contracted by the federal government, and state law enforcement vessels engaged in enforcement or search and rescue activities although some of these vessels may be subject to speed reductions under other conditions of permit. The simulation assumed all non-exempt vessels adhered to the simulated speed reductions. Vessel strike mortality was re-calculated in a scenario we refer from here on as the “slow-all” scenario. The results from the slow-all scenario were then compared to the “real-world” scenario, i.e., the estimated vessel strike mortality from unmodified real-world AIS vessel traffic data.

## Results

### Right whale distribution

**Fig. 4:**
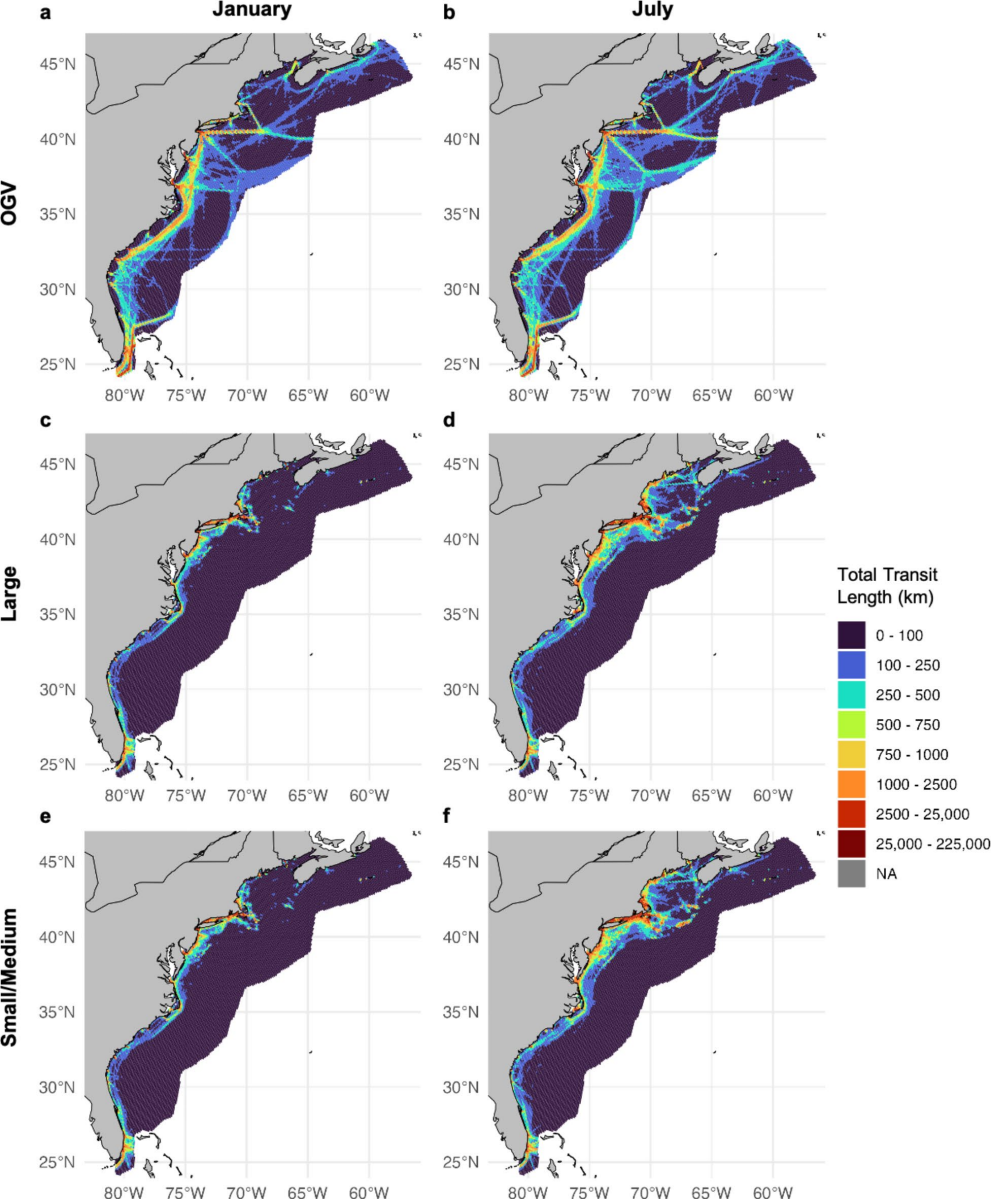
OGV, Large, and Small/Medium vessel traffic in two example months: January and July. The top two figures show average yearly total transit length by cell in (**a** ) January and (**b**) July for OGVs. The middle two figures show average yearly total transit length by cell in (**c**) January and (**d**) July for Large-sized vessels. The bottom two figures show average yearly total transit length by cell in (**e**) January and (**f**) July for Small/Medium-sized vessels. For Small/Medium-sized vessels, these maps do not include the correction factor applied to the number of interactions between whales and vessels within the encounter risk model. This figure was generated in R version 4.2.3 (https://www.r-project.org/). Country-level boundaries were sourced from Massicotte P, South A (2024). rnaturalearth: World Map Data from Natural Earth. R package version 1.0.

The right whale density model estimated high densities of right whales in the Mid-Atlantic and in southern New England and Cape Cod Bay during winter months, and in particular, predicted highest densities of right whales in U.S. waters during the months of November through April, as compared to summer months when a substantial fraction of the population migrated to Canadian waters (Fig. 4). Importantly, the right whale density model used here, version 12, better differentiated densities predicted within Mid-Atlantic waters during cooler months, compared to the version 9 model used in Garrison et al.^30^. The version 9 model lacked the covariates needed to distinguish the southern New England region, where numerous sightings were made over the past decade^51^, from areas further south, where right whales were present in lower density. The version 12 model used here provided density surfaces on a monthly time step corrected for effort and bias, and thus our composite vessel strike encounter risk model now more accurately reflects the localized high-density levels within Nantucket shoals during the winter (Fig. 4). Other differences and improvements found in version 12 of the model are described in full in Roberts et al.^37^.

### Right whale vertical behavior

We analyzed 146 depth-recording tags to evaluate differences in vertical behavior across right whale habitat range (Table S1, Table S2, Fig. 3a). This analysis revealed differences in time spent in the upper 5 and 15 m of the water column by geographic region (Fig. 3b). Right whales spent more time at or near the water surface in the Southeast, Cape Cod Bay, and Mid-Atlantic as compared to the Northeast. In Cape Cod Bay, we found right whales spent a weighted average of 95.3% and 91.0% of time above 15 and 5 m, respectively. In the Southeast, we found right whales spent a weighted average of 93.0% and 56.4% of time above 15 and 5 m, respectively. In contrast, in the Mid-Atlantic, we found right whales to spend a weighted average of 96.9% and 87.6% of time above 15 and 5 m, respectively. In the Northeast, we found right whales to spend an average of 58.7% and 39.2% of time above 15 and 5 m, respectively (Table S1, Fig. 3b).

### Vessel transit data

**Fig. 5:**
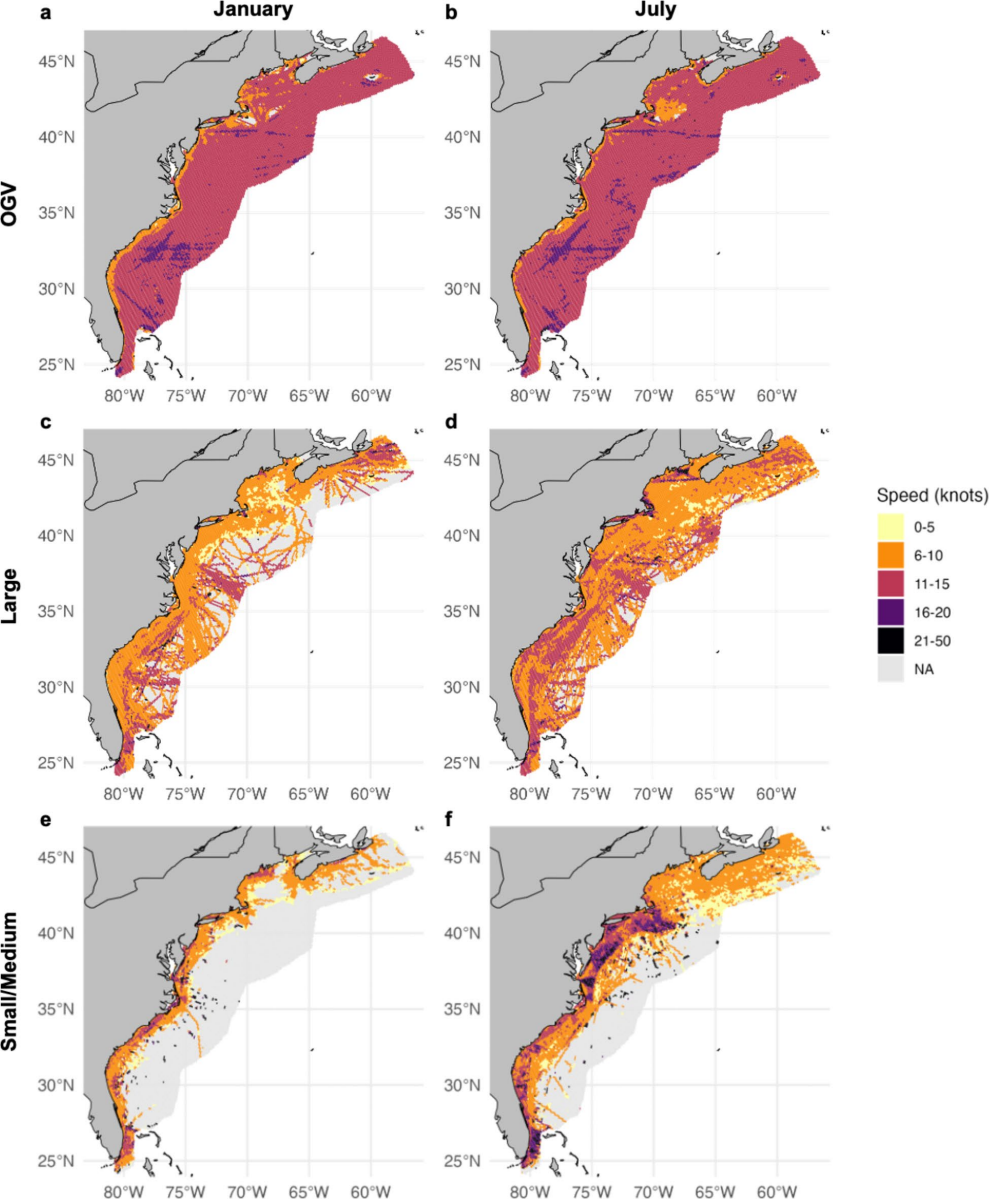
OGV, Large, and Small/Medium vessel traffic speeds in two example months: January and July. The top two figures show the mean speed per grid cell averaged over the study period in (**a**) January and (**b**) July for OGVs. The middle two figures show the mean speed per grid cell averaged over the study period in (**c**) January and (**d**) July for Large-size vessels. The bottom two figures show the mean speed per grid cell averaged over the study period in (**e**) January and (**f**) July for Small/Medium-size vessels. This figure was generated in R version 4.2.3 (https://www.r-project.org/). Country-level boundaries were sourced from Massicotte P, South A (2024). rnaturalearth: World Map Data from Natural Earth. R package version 1.0.

Within the model, we analyzed vessel traffic and resulting risk by three separate size classes: Small/Medium (26–65 feet), Large (65–350 feet), and OGVs (> 350 feet). Vessel traffic patterns for OGVs remained consistent within and among years, concentrated largely in the shipping lanes to major U.S. ports along the eastern seaboard, and in particular over the continental shelf (Fig. 2a, b). A large proportion of traffic in this size class was greater than 10 knots, particularly offshore and across the continental shelf (Fig. 5a, b). Large-sized vessels were broadly distributed over the continental shelf and less tied to specific shipping lanes and port areas, but included high densities of traffic in shipping lanes off of New York and Chesapeake Bay, as well as ferry routes in Cape Cod Bay, Martha’s Vineyard, Nantucket Shoals, Massachusetts Bay, Block Island, and Rhode Island (Fig. 2c, d). This size class accounted for the majority of transit lines (i.e., transit length in kilometers per cell). Average speeds for this class were lower in the winter months (e.g., January; Fig. 5c) than the summer months (e.g., July; Fig. 5d). Small/Medium-sized vessels peaked in summer months from May to September (e.g., July; Fig. 2f) along U.S. coastlines as compared to winter months (e.g., January; Fig. 2e). Higher densities of Small/Medium traffic were observed around ports and inlets. This traffic often operated at higher speeds than the other two size classes (Fig. 5e, f), but made up a smaller amount of total trackline relative to the other two size classes (4e, f). Importantly, however, this comparison was only made among the non-scaled AIS data, as the correction factor was only applied to this size class within the number of interactions between whales and vessels calculated within the encounter risk model and not the raw AIS data itself. Traffic generally occurred closer to shore, however was more broadly distributed and at higher densities during warmer months (Fig. 2f). Vessels in the Small/Medium size class are not required to carry AIS devices and are therefore underrepresented in AIS data and subsequent analyses. As detailed in the Methods, we applied a correction factor to vessel traffic within this size class to account for this. The correction factor applied increased vessel traffic in this category by ~ 3.5 times compared to what was documented originally in the AIS data.

### Encounter risk model results

**Fig. 6:**
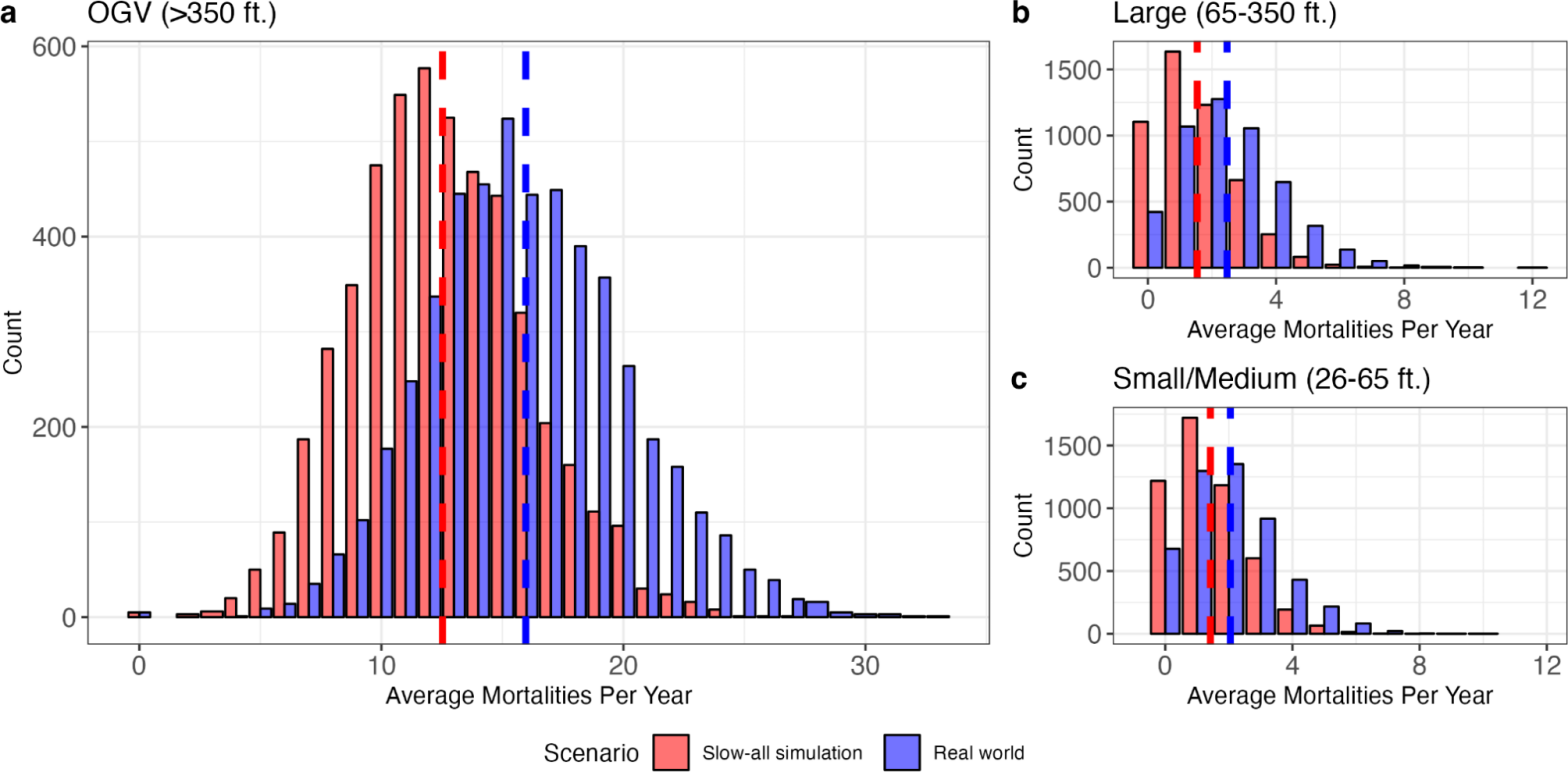
Bar plots of bootstrapped modeled average annual mortality rate (the sum of mortalities across all spatial cells and all months in each year, averaged across the 5 years included in the study, i.e., average number of whales killed per year) due to vessel strike from vessels under a real-world and slow-all scenario simulation of whale-vessel interactions with (**a**) OGVs, (**b**) Large-sized vessels, and (**c**) Small/Medium-sized vessels. Dotted lines indicate the mean of each scenario. All analyses were based on a population of 350 right whales^65^. This figure was generated in R version 4.2.3 (https://www.r-project.org/).

We calculated the number of lethal vessel strikes of right whales in U.S. waters based on the distribution and density of right whales and vessel traffic and several other whale and vessel-specific model parameters. The encounter risk model results revealed a large disparity between OGVs and the other two size classes in the percent contribution to total existing risk, as measured by estimated vessel strike mortality. The estimated average cumulative coastwide yearly rate of mortality for OGVs was 15.96 individuals per year, making up a 77.92% contribution to total risk (Table 1; Fig. 6a). The comparative estimated rate for Large-size vessels was 2.47 individual mortalities per year, making up 12.07% of total risk (Table 1; Fig. 6b). With the correction factor applied, the estimated rate for Small/Medium vessels averaged 2.05 mortalities per year, making up 10.01% of total risk (Table 1; Fig. 6c). Levels of whale mortality are largely consistent among years within each vessel size class (Figure S5-S10). Mortality under the real-world scenario increased slightly each year for the Small/Medium size class (Figure S5c), whereas mortality varied slightly but did not show an overall increase under the real-world scenario for OGVs and Large vessels (Figure S5a, b), though this also may be a function of slow increases in AIS adoption for vessels within these size classes, as the correction factor was informed by only one year of registration data.

**Fig. 7:**
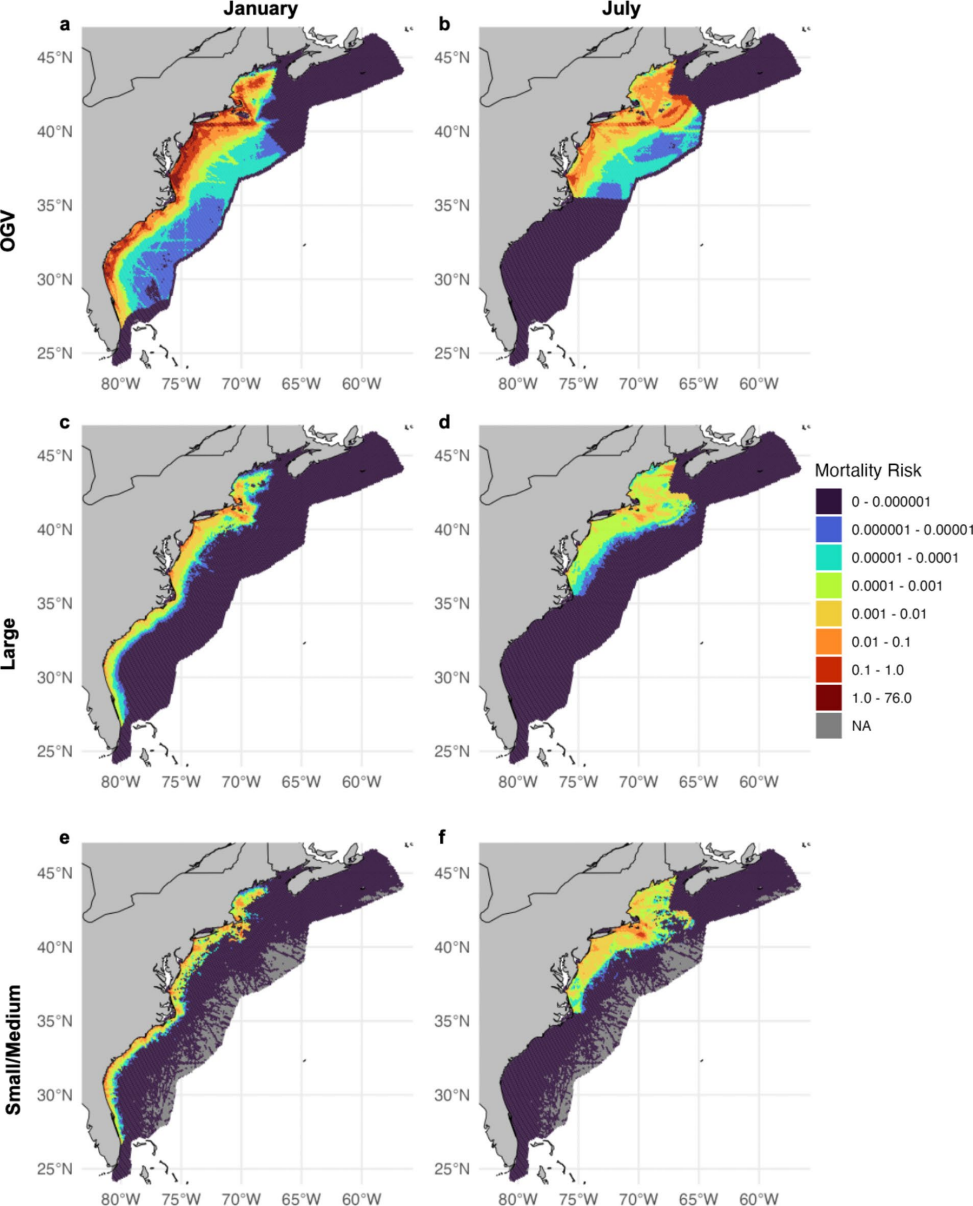
Proportion of total annual vessel strike mortality risk by spatial cell for (**a**) OGVs in January, (**b**) OGVs in July, (**c**) Large-sized vessels in January, (**d**) Large-sized vessels in July, (**e**) Small/Medium-sized vessels in January, and (**f**) Small/Medium-sized vessels in July. All plots show the 5-year average of each spatial cell from 2017–2022, excluding 2020 within each respective month. A linear- scaling transformation was also applied to the data to better show differences in cell values among months and vessel size classes. All analyses were based on a population of 350 right whales^65^. This figure was generated in R version 4.2.3 (https://www.r-project.org/). Country-level boundaries were sourced from Massicotte P, South A (2024). rnaturalearth: World Map Data from Natural Earth. R package version 1.0.

Mortality risk was concentrated in areas associated with high densities of both whales and vessels (Fig. 7). In winter months such as January, when right whales are distributed throughout U.S. waters, risk was also dispersed throughout coastal areas of the U.S. East Coast (Fig. 7a, c, & e), while in summer months such as July, risk was more heavily concentrated in the northern area of the right whale’s range within the Gulf of Maine and Nantucket Shoals (Fig. 7b, d, & f). During winter months, we identified high mortality risk areas throughout the Southeast, along the Mid-Atlantic coast, south of Martha’s Vineyard and Nantucket, and in Cape Cod Bay particularly from the Large-sized vessels. Additionally, Small/Medium-sized vessels present a similar mortality level to the Large-sized class in nearshore waters all along the coast, particularly, in Cape Cod Bay and south of Nantucket as well as the Southeast (Fig. 7e, f). In summer and early fall, we found that mortality risk was generally lower across the U.S. coast. The area of highest mortality risk for the large size class was in Cape Cod Bay and South of Nantucket (Fig. 7c, d), whereas the OGV size class was responsible for most mortality risk in the Mid-Atlantic (Fig. 7a, b).

The “slow-all” simulation, where all vessels were slowed to approximately 10 knots, allowed us to evaluate the utility of implementing broad speed restrictions for all vessels. Within the slow-all scenario, the mean annual rate of mortality dropped to 12.52 individuals/year (21.54% reduction) for OGVs, an average of 1.54 mortalities/year (37.68% reduction) for Large-sized vessels, and an average of 1.42 mortalities/year (30.71% reduction) for Small/Medium-sized vessels (Table 1; Fig. 7). When weighted by the percent of total risk contributed by each size class (Table 1, “Percent of total risk”), we estimate that the overall weighted average of risk reduced by slowing all vessels to 10 knots is 24.5%. Moreover, the distribution of bootstrap results for the slow-all scenario was skewed towards lower levels of mortality, while the distribution for the real-world scenario was skewed towards higher levels of mortality (Table 1; Fig. 6).

**Table 1:** Average annual mortality estimates by vessel size class.

Size class	Percent of total risk	Metric	Real-world	Slow-all simulation
OGV (> 350 ft.)	77.92%	mean	15.96	12.52
		sd	4.15	3.59
		median	16.0	12.0
		decrease in mean risk	--	21.54%
Large (65–350 ft.)	12.07%	mean	2.47	1.54
		sd	1.60	1.26
		median	2.0	1.0
		decrease in mean risk	--	37.68%
Small/Medium (26–65 ft.)	10.01%	mean	2.05	1.42
		sd	1.46	1.20
		median	2.0	1.0
		decrease in mean risk	--	30.71%

Cumulative risk is calculated across the study area each year, and averaged by year to receive average annual mortality estimates for each vessel size class. Percent decreases in mortality between scenarios are calculated using means.

## Discussion

In this study, we improve upon an existing vessel strike encounter risk model framework, as described in Martin et al.^19^, which has previously been modified to conduct several vessel strike risk assessments (e.g.,^28–30,52^. We build on these approaches by 1) scaling the model to a reasonable population size rather than relying on density from the model and calculating the number of individual mortalities estimated per year, 2) incorporating an updated and more sophisticated avoidance function 3) performing a sensitivity analysis to gauge the level of uncertainty contributed by each model parameter, 4) incorporating a new probability of lethality curve^24^ and 5) refining the parameterization to better reflect differences between vessel size classes within both the lethality curve and drafts that are more specific to vessel sizes. Here, we also incorporate up-to-date vessel traffic data collected via AIS transceivers, as well as the best available empirical and modeled data on right whale habitat distribution and vertical habitat use. These improvements provide more refined estimates of annual mortality for right whales along the U.S. East Coast and also allow for estimated proportions of risk attributed to individual vessel size classes. As a result, this model provides an opportunity to predict the impact of more targeted management strategies.

Here, we estimate that the annual mortality of right whales due to vessel strike in U.S. waters (~ 17.5 whales/year, Table 1; Fig. 6, Table S4) is nearly an order of magnitude higher than the number of documented right whale deaths and serious injuries in U.S. waters, which average 2.4 whales per year^7,8^. This is not surprising given that many vessel strike related deaths and serious injuries go unobserved. In fact, our estimated yearly mortality rate is within the range of what we would expect based on the Pace et al.^11^mark-recapture model and estimated death, and the apportionment used for the population vulnerability assessment (PVA) in Linden^8^. Linden^8^calculated an average of 22.25 deaths per year from 2017 to 2022, ranging from an average of 7.88 deaths in 2020–2021 to 42.92 deaths in 2017–2018. Similar to our results, these modeled results of mortality from the PVA, which include all deaths (e.g., death related to vessel strike, death related to entanglement, and natural mortality) are much higher than the number of documented right whale deaths and lethal/serious injuries in U.S. waters^7,8^. Vessel strikes at the level estimated here are likely to contribute to the rapid decline and possible extinction of this critically endangered species. Without substantial reduction in vessel strikes and entanglement risk, this species will continue to be on this trajectory.

Regional differences in right whale vessel strike mortality risk were evident from this analysis, which is largely dependent on the amount of time whales spend at the surface in each region. As a migratory species, right whales engage in different behaviors along the U.S. East Coast, influencing the amount of time spent within the strike zone of operating vessels within each habitat area^15,53,54^. As Garrison et al.^30^indicated, right whales are particularly vulnerable to vessel strikes in the Mid-Atlantic as they spend a large portion of time in the upper part (0 to 16 m) of the water column while migrating and occasionally foraging^55^. Here we also identify disproportionate risk in Cape Cod Bay, where right whales often engage in surface-feeding behavior and spend 91.0% of time above 5 m, and vessel traffic is dense and at relatively high speeds across vessel size classes. Our surface time estimates are higher than those reported in Ganley et al.^56^who found an average of 34% surface time from January through April in the Cape Cod Bay region based on a survey of focal follows. We suggest this is likely due to the fact that right whales have been observed to spend significant time just below the water surface, where they cannot be seen but continue to feed on prey within the upper 5 m of the water column^44^. Importantly, Ganley et al.^56^ found a significant level of variability in surface time in Cape Cod Bay, ranging from an average of 16% in January to an average of 55% in April. The model presented here does not currently account for this scale of monthly variability and would be improved by finer resolution of variation in dive behavior across regions and life-history phases, as data availability allows.

The vessel strike encounter risk model and results presented here improve our ability to estimate mortality due to vessel strike by vessel size class. Outputs under both the real-world and slow-all scenario indicate that OGVs pose the greatest risk to right whales. In general, longer vessels have deeper drafts, and the weight (e.g., cargo load) of a vessel can impact how deep it sits in the water^57^. The draft of a vessel can influence strike risk to whales both because deeper drafts overlap more in space with the depth range of whales and because deeper drafts make it more difficult for a whale to evade a strike as they must dive deeper to avoid an oncoming vessel. Larger vertical “risk zones”, as a result of draft and propellor suction depth, also creates unavoidable zones when OGVs travel in waters of depth less than 33 m (i.e., the draft plus propeller suction depth plus average height of a whale and whales have nowhere to dive and escape). Finally, the OGV size class is characterized by a significantly heightened probability of a lethal outcome, should a strike occur^24^. Reducing speed was more effective at reducing the lethality of a strike for vessels under 350 feet as compared to those over 350 feet within the slow-all simulation. While we demonstrate that slowing vessel speeds reduced mortality risk regardless of vessel size class, slowing vessels was more effective for the Large and Small/Medium size classes as compared to OGVs (Table 1; Fig. 6), likely because the increased draft and propeller suction depth for OGVs make them more difficult to avoid at any speed, and a vessel strike from an OGV traveling at any speed is highly likely to be lethal^24^.

As a result, the OGV size class contributes nearly 78% of mortalities according to these results, however, uncertainty that we have fully captured vessel strike risk of smaller size classes remains due to the lack of AIS data for these vessels, despite inclusion of the correction factor. Over the last 24 years, 32% of reported lethal strikes involved vessels under 65 feet. However, here we predict that vessels in the small/medium size class account for only 10% of lethal strikes. Although this isn’t an “apples to apples’’ comparison, the difference between observed and modeled results is likely attributable to the fact that OGV collisions with large whales, such as right whales, go largely undetected and OGVs also spend more time further offshore where a carcass is less likely to be found or wash up on shore, resulting in a high number of cryptic (i.e., unrecorded) mortalities^11,25^, whereas smaller vessels are often more aware that they have collided with a whale. Consequently, we suggest that reported strikes may be biased towards smaller vessels and OGVs may be involved in a much higher number of collisions than are currently reported.

While this study improves upon previous vessel strike encounter risk models, there are still several significant sources of uncertainty within the model and assumptions that were required (Supplementary S2, Table S5). The sensitivity analysis performed indicates which parameters have the largest effect on resulting mortality and specifies how changes in parameters increase or decrease estimates of mortality (Supplementary Section S2, Table S4, Figure S3). While measures of central tendency (e.g., mean and median, Table 1) are useful for understanding the impact of changes to status quo conditions, bootstrapped results of mortality still showed high variation (Fig. 6). We used empirical data to calculate the proportion of time that a whale spends at the water surface or in shallow depths; however, we lacked tags deployed on right whales in the Mid-Atlantic specifically (Table S1). It is also likely that vertical behavior varies by life history phase (e.g., mom-calf versus adult male) and on a finer temporal resolution (i.e., seasonally) than we had tag data to explore (Table S2). Additionally, risk calculations would ideally be based on actual vessel drafts recorded within the AIS data; however, we found a very limited number of vessel records in the AIS data contain draft attributes (Figure S4). Further, based on Silber et al.^50^, we apply a simple scalar to account for propeller suction depth in vessels > 350 feet in length. It is likely that vessels below 350 feet al.so have some level of propeller suction; however, the degree of suction likely varies by vessel type. Future vessel strike risk analyses would greatly benefit from draft and propeller suction depth levels that account for both vessel size on a continuous scale and vessel type (e.g., sailing versus cargo vessels).

The mean monthly density predictions for 2010–2019 from Roberts et al.^37^ used here for understanding right whale distribution are considered the best available data on right whale density along the U.S. East Coast. However, right whale density and distribution varies inter-annually within this region. Future iterations of this risk model could incorporate variability and uncertainty in right whale density and distribution to better understand how this influences vessel strike risk. In addition, while forecasts of future right whale distributions are not currently available, they could be particularly useful in predicting future vessel strike risk assuming relatively constant vessel traffic patterns, though corresponding future vessel traffic predictions would be ideal.

The most significant source of model uncertainty fell within the whale avoidance function (Supplementary S2, Figure S1, Table S4). Whereas Garrison et al.^30^simulated horizontal movement in a direction of 0–90 degrees from the vessel track, here we simulate a vertical descent, which may be a more realistic reaction of a right whale to an oncoming vessel according to limited evidence^58,59^. Right whale behavior is different from that of blue whales, but by using descent rates recorded from right whales, we believe this avoidance function is appropriate for use here. Recently, studies have aimed to fill the knowledge gap as to whether or not whales actively avoid oncoming vessels; however, these studies are often conducted on humpback whales^60,61^. Garrison et al.^24^ demonstrated a significant difference between humpbacks and other large whale species in relation to lethal vessel strike, so while these recent studies on humpback whale avoidance are important to consider, the results of Garrison et al.^24^, suggest it is not appropriate to use data from these studies to inform avoidance for right whales.

Overall, little data exist on whether, or how, right whales may modify their behavior to explicitly avoid oncoming vessels. Here we show that if there is no vessel avoidance behavior by whales, in situations where whales and vessels are in immediate proximity, modeled vessel strike mortality would be much higher than what is realistic based on independent methods to estimate overall right whale mortality following the methods of Linden^8^ and Pace et al.^11^ (Figure S1, Table S4, Figure S3). We suggest that the frequency of whale avoidance, given immediate proximity, is almost certainly greater than 0%; however, at present there is no empirical basis for estimating the true frequency of vessel avoidance for any large whale species. Therefore, it is useful to consider the results of both scenarios from our sensitivity analysis as bounding estimates around the true value. The likelihood of active vessel avoidance by whales may also be related to whale behavioral state, such as if whales are surface active, foraging, or resting, as well as the whale’s life history phase, such as mom-calf pairs, adults, and juveniles. Further, it remains unclear at what ranges and under what scenarios (i.e., vessel approach speed) a whale may determine there is a need to modify behavior, given their maneuverability capabilities. Whales may also broadly modify their behavioral posture in response to areas of high traffic volume, such as moving more quickly, or at deeper depths through high traffic areas such as across shipping channels. Very little is known about these types of behaviors and their effects on avoidance probability; therefore, this remains an important data gap to be addressed in future encounter risk models.

Accurately assessing risk of smaller vessels (i.e., less than 65 ft) to whales in U.S. waters has been a long-standing issue in vessel strike encounter risk models and analyses^5,30^given that USCG AIS carriage requirements do not apply to most vessels < 65 ft (19.8 m) in length (80 FR 5281, January 30, 2015; 80 FR 2050, April 7, 2016). Here, to supplement the available AIS data, we applied a correction factor to the Small/Medium size class using USCG Certificate of Documentation (COD) vessel registration and NMFS Fishing Permit data records. With inclusion of the correction factor, Small/Medium vessels contributed risk on the same order of magnitude as the Large size class, suggesting that the risk posed by this Small/Medium size class should not be overlooked. However, this model does not take into account the higher level of maneuverability of small boats - where these vessels are much more likely to make frequent changes in speed and direction^5,25^. It is important to continue to develop quantitative and more refined methods to assess the impact of smaller vessels on vessel strike risk and mortality to large whales.

Despite existing data gaps and uncertainty, the model presented here improves upon previous iterations of vessel strike encounter risk models, and importantly, can be used to estimate the benefit of various management strategies. In the future, we aim to adapt this model to a more generalized form to be used for other species and systems and ultimately develop tools for wide-spread use by the marine science community. North Atlantic right whales are one of the most data-rich large whale species which allows us to validate our results via comparison to estimates of mortality from independent methods based on tracking individuals in the population; therefore, the results of strike rate models fit to this system may be valuable for other more data-poor species of concern. When applying this model to other scenarios, it may be useful to select model parameter inputs based on the desired conservatism of mortality estimates. Here, we selected values for each model parameter based on the best available empirical data, while ensuring the modeled mortality was comparable to mortality estimates from other, independent studies that rely on the tracking of individual whales^8,11^.

As the oceans continue to industrialize and changes to vessel traffic increase due to activities such as offshore wind energy and aquaculture development, this vessel strike encounter risk model can be used as a tool to evaluate the spatial and temporal boundaries of potential management efforts and mitigation measures to reduce vessel strike risk to large whales, while minimizing economic impacts. Currently employed measures typically include altering vessel routes and voluntary and/or mandatory vessel speed restrictions, but our modeling framework could also be used to evaluate the effectiveness of new technologies designed to reduce vessel strike risk by including additional terms in the model, such as the probability a vessel detects and successfully avoids an encounter with a whale. It’s also important to consider current and potential changes in the distributions and movements of large whales as climate change continues to alter the biophysical makeup of critical ocean habitats. Anticipating large shifts in habitat use, such as those detected for right whales post-2010^38,62^, by integrating potential changes in distribution patterns into an encounter risk model may help prevent unexpected increases in human-caused mortality that can be detrimental to vulnerable populations^63,64^. While this vessel strike encounter risk model can provide estimates of risk reduction, the ultimate measure of effectiveness will be whether or not strikes are actually reduced, a change that will require continued population monitoring. Through adaptive management, this model could be used to make predictions about potential effects of a management application, which can then be applied and monitored for effectiveness, followed by an evaluation of the potential need for additional management measures once post implementation data are available to inform an updated model.

## Electronic supplementary material

Below is the link to the electronic supplementary material.

Supplementary Material 1

## Data Availability

The datasets used in this study are available from the corresponding author upon reasonable request. The North Atlantic right whale density surfaces are available at: https://seamap.env.duke.edu/models/Duke/EC/EC_North_Atlantic_right_whale_history.html. The vessel strike encounter risk model and code is available at: https://github.com/SEFSC/VesselStrikeRiskModel.

## References

[1] Pirotta, V., Grech, A., Jonsen, I. D., Laurance, W. F. & Harcourt, R. G. Consequences of global shipping traffic for marine giants. *Front. Ecol. Environ.* **17**, 39–47. 10.1002/fee.1987 (2019).

[2] Nyhus, P. J. Human-Wildlife Conflict and Coexistence. *Annu. Rev. Environ. Resour.* **41**, 143–171 (2016).

[3] Halpern, B. S. et al. Spatial and temporal changes in cumulative human impacts on the world’s ocean. *Nat. Commun.* **6**10.1038/ncomms8615 (2015). 10.1038/ncomms8615 PMC4510691 26172980

[4] Calambokidis, J. et al. Differential vulnerability to ship strikes between Day and Night for Blue, Fin, and Humpback whales based on Dive and Movement Data from Medium Duration Archival Tags. *Front. Mar. Sci.* **6**10.3389/fmars.2019.00543 (2019).

[5] Schoeman, R. P., Patterson-Abrolat, C. & Plön, S. A global review of vessel collisions with marine animals. *Front. Mar. Sci.* **7**10.3389/fmars.2020.00292 (2020).

[6] Pirotta, E. et al. Estimating the effects of stressors on the health, survival and reproduction of a critically endangered, long-lived species. *Oikos***e09801**10.1111/oik.09801 (2023).

[7] Hayes, S. U. S., Atlantic & Gulf of Mexico Marine Mammal Stock Assessments. and North Atlantic Right Whale (*Eubalaena glacialis*): Western Atlantic Stock. (2023). https://www.fisheries.noaa.gov/s3/2023-08/North-Atlantic-Right-Whale-Western-Atlantic-2022.pdf

[8] Linden, D. W. Population size estimation of North Atlantic right whales from 1990–2023. (2024). 10.25923/bjn8-kx95

[9] Endangered Species Act of. 16 U.S.C. § 1531–1544 (2024). (1973).

[10] Cooke, J. G. *Eubalaena glacialis* (errata version published in 2020). The IUCN Red List of Threatened Species 2020: e.T41712A178589687. 10.2305/IUCN.UK.2020-2.RLTS.T41712A178589687.en

[11] Pace, R. M., Williams, R., Kraus, S. D., Knowlton, A. R. & Pettis, H. M. Cryptic mortality of North Atlantic right whales. *Conserv. Sci. Pract.* **3**10.1111/csp2.346 (2021).

[12] Derville, S., Torres, L. G., Zerbini, A. N., Oremus, M. & Garrigue, C. Horizontal and vertical movements of humpback whales inform the use of critical pelagic habitats in the western South Pacific. *Sci. Rep.* **10**10.1038/s41598-020-61771-z (2020). 10.1038/s41598-020-61771-z PMC7078318 32184421

[13] Hazen, E. L. et al. Fine-scale prey aggregations and foraging ecology of humpback whales *Megaptera novaeangliae*. *Mar. Ecol. Prog Ser.* **395**, 75–89. 10.3354/meps08108 (2009).

[14] Abrahms, B. et al. Memory and resource tracking drive blue whale migrations. *Proc. Natl. Acad. Sci.* **116**, 5582–5587. 10.1073/pnas.1819031116 (2019). 30804188 10.1073/pnas.1819031116 PMC6431148

[15] Gowan, T. A. et al. Temporal and demographic variation in partial migration of the North Atlantic right whale. *Sci. Rep.* **9**10.1038/s41598-018-36723-3 (2019). 10.1038/s41598-018-36723-3 PMC6344554 30674941

[16] Baumgartner, M. F., Cole, T. V. N., Campbell, R. G., Teegarden, G. J. & Durbin, E. G. Associations between North Atlantic right whales and their prey, *Calanus finmarchicus*, over diel and tidal time scales. *Mar. Ecol. Prog Ser.* **264**, 155–166. 10.3354/meps264155 (2003).

[17] Baumgartner, M. F., Lysiak, N. S. J., Schuman, C., Urban-Rich, J. & Wenzel, F. W. Diel vertical migration behavior of *Calanus finmarchicus* and its influence on right and sei whale occurrence. *Mar. Ecol. Prog Ser.* **423**, 167–184. 10.3354/meps08931 (2011).

[18] National Marine Fisheries Service (NMFS). *North Atlantic Right Whale (Eubalaena glacialis) Vessel Speed Rule Assessment* (National Marine Fisheries Service, Office of Protected Resources, 2020).

[19] Martin, J. et al. A quantitative framework for investigating risk of deadly collisions between marine wildlife and boats. *Methods Ecol. Evol.* **7**, 42–50. 10.1111/2041-210X.12447 (2016).

[20] Redfern, J. V. et al. Evaluating stakeholder-derived strategies to reduce the risk of ships striking whales. *Divers. Distrib.* **25**, 1575–1585. 10.1111/ddi.12958 (2019).

[21] Blondin, H., Abrahms, B., Crowder, L. B. & Hazen, E. L. Combining high temporal resolution whale distribution and vessel tracking data improves estimates of ship strike risk. *Biol. Conserv.* **250**10.1016/j.biocon.2020.108757 (2020).

[22] Conn, P. B. & Silber, G. K. Vessel speed restrictions reduce risk of collision-related mortality for North Atlantic right whales. *Ecosphere* **4**, 1–16. 10.1890/ES13-00004.1 (2013).

[23] Kelley, D. E., Vlasic, J. P. & Brillant, S. W. Assessing the lethality of ship strikes on whales using simple biophysical models. *Mar. Mamm. Sci.* **37**, 251–267. 10.1111/mms.12745 (2021).

[24] Garrison, L. P. et al. The effects of vessel speed and size on the lethality of strikes of large whales in U.S. waters. *Front. Mar. Sci*. (In Press).

[25] Laist, D. W., Knowlton, A. R., Mead, J. G., Collet, A. S. & Podesta, M. COLLISIONS BETWEEN SHIPS AND WHALES. *Mar. Mamm. Sci.* **17**, 35–75. 10.1111/j.1748-7692.2001.tb00980.x (2001).

[26] Moore, M. J., Mitchell, G. H., Rowles, T. K. & Early, G. Dead cetacean? Beach, bloat, float, sink. *Front. Mar. Sci.* **7**10.3389/fmars.2020.00333 (2020).

[27] Moore, M. J. et al. Criteria and case definitions for serious injury and death of pinnipeds and cetaceans caused by anthropogenic trauma. *Dis. Aquat. Org.* **103**, 229–264. 10.3354/dao02566 (2013). 10.3354/dao02566 23574708

[28] Rockwood, R. C., Calambokidis, J. & Jahncke, J. High mortality of blue, humpback and fin whales from modeling of vessel collisions on the U.S. West Coast suggests population impacts and insufficient protection. *PLoS One*. **12**, e0183052. 10.1371/journal.pone.0183052 (2017). 28827838 10.1371/journal.pone.0183052 PMC5565115

[29] Crum, N., Gowan, T., Krzystan, A. & Martin, J. Quantifying risk of whale–vessel collisions across space, time, and management policies. *Ecosphere* **10**, e02713. 10.1002/ecs2.2713 (2019).

[30] Garrison, L. P., Adams, J., Pattterson, E. M. & Good, C. P. *NOAA Technical Memorandum NMFS-SEFSC-757 Assessing the risk of Vessel Strike Mortality in North Atlantic* (right whales along the U.S. East Coast, 2022).

[31] Redfern, J.V., Hodge, B. C., Pendleton, D. E., Knowlton, A. R., Adams, J., Patterson,E. M., … Roberts, J. J. Estimating reductions in the risk of vessels striking whales achieved by management strategies. Biol. Cons.290, 110427 (2024). http://dx.doi.org/10.1016/j.biocon.2023.110427.

[32] Keen, E. M. et al. Ship-strike forecast and mitigation for whales in Gitga’at First Nation territory. *Endanger. Species Res.* **51**, 31–58. 10.3354/esr01244 (2023).

[33] Record, N. R., Runge, J. A., Pendleton, D. E., Balch, W. M., Davies, K. T., Pershing,A. J., … Thompson, C. R. Rapid climate-driven circulation changes threaten conservation of endangered North Atlantic right whales. Oceanography32, 162–169. (2019). http://dx.doi.org/10.5670/oceanog.2019.201.

[34] Meyer-Gutbrod, E. L., Greene, C. H., Davies, K. T. & Johns, D. G. Ocean regime shift is driving collapse of the North Atlantic right whale population. *Oceanography* **34**, 22–31. 10.5670/oceanog.2021.308 (2021).

[35] Meyer-Gutbrod, E. L. et al. Redefining North Atlantic right whale habitat-use patterns under climate change. *Limnol. Oceanogr.* **68**, 71–S86. 10.1002/lno.12242 (2023).

[36] O’Brien, O. et al. Repatriation of a historical North Atlantic right whale habitat during an era of rapid climate change. *Sci. Rep.* **12**10.1038/s41598-022-16200-8 (2022). 10.1038/s41598-022-16200-8 PMC9300694 35859111

[37] Roberts, J. J. et al. North Atlantic right whale density surface model for the US Atlantic evaluated with passive acoustic monitoring. *Mar. Ecol. Prog Ser.* **732**, 167–192. 10.3354/meps14547 (2024).

[38] Davis, G. E. et al. Long-term passive acoustic recordings track the changing distribution of North Atlantic right whales (*Eubalaena glacialis*) from 2004 to 2014. *Sci. Rep.* **7**10.1038/s41598-017-13359-3 (2017). 10.1038/s41598-017-13359-3 PMC5647423 29044130

[39] United States Coast Guard (USCG). Merchant Vessels of the United States. (2021). https://www.dco.uscg.mil/Our-Organization/Assistant-Commandant-for-Prevention-Policy-CG-5P/Inspections-Compliance-CG-5PC-/Office-of-Investigations-Casualty-Analysis/Merchant-Vessels-of-the-United-States/

[40] March, D., Metcalfe, K., Tintoré, J. & Godley, B. J. Tracking the global reduction of marine traffic during the COVID-19 pandemic. *Nat. Comm.* **12**10.1038/s41467-021-22423-6 (2021). 10.1038/s41467-021-22423-6 PMC8079689 33907197

[41] Millefiori, L. M. et al. COVID-19 impact on global maritime mobility. *Sci. Rep.* **11**10.1038/s41598-021-97461-7 (2021). 10.1038/s41598-021-97461-7 PMC8433355 34508144

[42] S&P Global Market Intelligence. Sea-web™: the ultimate marine online database. (2023). https://www.spglobal.com/marketintelligence/en/mi/products/sea-web-maritime-reference.html

[43] Dombroski, J. R. G., Parks, S. E. & Nowacek, D. P. Dive behavior of North Atlantic right whales on the calving ground in the Southeast USA: implications for conservation. *Endang Species Res.* **46**, 35–48. 10.3354/esr01141 (2021).

[44] Parks, S. E. Dangerous dining: surface foraging of North Atlantic right whales increases risk of vessel collisions. *Biol. Lett.* **8**, 57–60. 10.1098/rsbl.2011.0578 (2012). 21813549 10.1098/rsbl.2011.0578 PMC3259960

[45] Baumgartner, M. F. & Mate, B. R. Summertime foraging ecology of North Atlantic right whales. *Mar. Ecol. Prog Ser.* **264**, 123–135. 10.3354/meps264123 (2003).

[46] Baumgartner, M. F., Wenzel, F. W., Lysiak, N. S. J. & Patrician, M. R. North Atlantic right whale foraging ecology and its role in human-caused mortality. *Mar. Ecol. Prog Ser.* **581**, 165–181. 10.3354/meps12315 (2017).

[47] McKenna, M. F., Calambokidis, J., Oleson, E. M., Laist, D. W. & Goldbogen, J. A. Simultaneous tracking of blue whales and large ships demonstrates limited behavioral responses for avoiding collision. *Endang Species Res.* **27**, 219–232. 10.3354/esr00666 (2015).

[48] Moore, M. J., Knowlton, A. K., Kraus, S. D., McLellan, W. A. & Bonde, R. K. Morphometry, gross morphology and available histopathology in North Atlantic right whale (*Eubalaena glacialis*) mortalities (1970–2002). *J. Cetacean Res. Manage.* **6**10.47536/jcrm.v6i3.762 (2005).

[49] NOAA National Centers for Environmental Information. 2022: ETOPO 2022 15 Arc-Second Global Relief Model. (2022). 10.25921/fd45-gt74

[50] Silber, G. K., Slutsky, J. & Bettridge, S. Hydrodynamics of a ship/whale collision. *J. Exp. Mar. Biol. Ecol.* **391**, 10–19. 10.1016/j.jembe.2010.05.013 (2010).

[51] Quintana-Rizzo, E. et al. Residency, demographics, and movement patterns of North Atlantic right whales *Eubalaena glacialis* in an offshore wind energy development area in southern New England, USA. *Endang Species Res.* **45**, 251–268. 10.3354/esr01137 (2021).

[52] Rockwood, R. C., Adams, J., Silber, G. & Jahncke, J. Estimating effectiveness of speed reduction measures for decreasing whale-strike mortality in a high-risk region. *Endang Species Res.* **43**, 145–166. 10.3354/esr01056 (2020).

[53] Kenney, R. D., Mayo, C. A. & Winn, H. E. Migration and foraging strategies at varying spatial scales in western North Atlantic right whales: a review of hypotheses. *J. Cetacean Res. Manag (Spec Issue)*. **2**, 251–260. 10.47536/jcrm.vi.283 (2001).

[54] Brillant, S. W., Vanderlaan, A. S. M., Rangeley, R. W. & Taggart, C. T. Quantitative estimates of the movement and distribution of North Atlantic right whales along the northeast coast of North America. *Endang Species Res.* **27**, 141–154. 10.3354/esr00651 (2015).

[55] Whitt, A. D., Dudzinski, K. & Laliberté, J. R. North Atlantic right whale distribution and seasonal occurrence in nearshore waters off New Jersey, USA, and implications for management. *Endang Species Res.* **20**, 59–69. 10.3354/esr00486 (2013).

[56] Ganley, L. C., Brault, S. & Mayo, C. A. What we see is not what there is: estimating North Atlantic right whale *Eubalaena glacialis* local abundance. *Endang Species Res.* **38**, 101–113. 10.3354/esr00938 (2019).

[57] NOAA National Ocean Service. An Inch of Water. What’s it Worth?. (2017). oceanservice.noaa.gov/economy/inch-water/

[58] Nowacek, D. P. and J. M. P. and T. P. L. North Atlantic right whales (*Eubalaena glacialis*) ignore ships but respond to alerting stimuli. *Proc. R. Soc. B.* 271, 227–231 (2004). 10.1098/rspb.2003.2570 10.1098/rspb.2003.2570 PMC1691586 15058431

[59] Wiley, D. N. J. Vessel strike mitigation lessons from direct observations involving two collisions between noncommercial vessels and North Atlantic right whales (*Eubalaena glacialis*). *Mar. Mamm. Sci.* **32**10.1111/mms.12326 (2016).

[60] Gulesserian, M., Slip, D., Heller, G. & Harcourt, R. Modelling the behaviour state of humpback whales Megaptera novaeangliae in response to vessel presence off Sydney, Australia. *Endang Species Res.* **15**, 255–264. 10.3354/esr00380 (2011).

[61] Helm, J. E., Gende, S. M. & Lukacs, P. M. Quantifying behavior and collision risk of humpback whales surfacing near large ships: implications for detection and avoidance. *Endang Species Res.* **51**, 115–126. 10.3354/esr01246 (2023).

[62] Davies, K. T. A. et al. Variation in North Atlantic right whale *Eubalaena glacialis* occurrence in the Bay of Fundy, Canada, over three decades. *Endang Species Res.* **39**, 159–171. 10.3354/esr00951 (2019).

[63] Davies, K. T. A. & Brillant, S. W. Mass human-caused mortality spurs federal action to protect endangered North Atlantic right whales in Canada. *Mar. Policy*. **104**, 157–162. 10.1016/j.marpol.2019.02.019 (2019).

[64] Pettis, H. M., Pace, R. M. I. & Hamilton, P. K. *North Atlantic Right Whale Consortium 2020 Annual Report Card*. www.narwc.org (2021).

[65] Linden, D. Population size estimation of North Atlantic right whales from 1990-2022. 10.25923/3v2z-j845 (2023).

