# Supplementary material for: Vessel strike encounter risk model informs mortality risk for endangered North Atlantic right whales along the United States east coast
Source: Sci Rep. 2025 Jan 3;15:736. doi: 10.1038/s41598-024-84886-z (PMC11699073; doi:10.1038/s41598-024-84886-z)
Supplement: Supplementary file 1 — Supplementary Material 1 [file 41598_2024_84886_MOESM1_ESM.docx]

**Vessel strike encounter risk model informs mortality risk for endangered North Atlantic Right Whales along the United States East Coast**

**Hannah Blondin*^1,2^, Lance Garrison^2^, Jeff Adams^4^, Jason Roberts^3^, Caroline Good^4^, Meghan Gahm^4^, Niki E. Lisi^4^ , Eric Patterson^4^**

^1^ University of Miami University of Miami Cooperative Institute for Marine & Atmospheric Studies (CIMAS), Miami, FL, 33149, USA

^2^ NOAA Fisheries, Southeast Fisheries Science Center, Miami, FL, 33149, USA

^3^ Marine Geospatial Ecology Laboratory, Duke University, Durham, NC 27708, USA

^4^ NOAA Fisheries, Office of Protected Resources, Silver Spring, MD 20910, USA

***Corresponding author:**

Hannah Blondin (hannah.blondin@noaa.gov), National Marine Fisheries Service, Southeast Fisheries Science Center, Marine Mammal and Turtle Division, 75 Virginia Beach Dr., Miami, FL 33149

# **SUPPLEMENTARY MATERIALS**

**S1. Depth tags: additional information**

**Table S1: Depth-recording tags analyzed by geographic region. Known sex and age classes are listed as C=Calf, J=Juvenile, A=Adult, M=Male, F=Female. Unknown attributes are not listed and left blank.**

| **Analysis region** | **Tagging region** | **n** | **Tag type** | **Tag duration (hr)** | **Sex;**  **Age class** | **Data sources or data holder** |
| --- | --- | --- | --- | --- | --- | --- |
| Cape Cod Bay | Cape Cod Bay | 16 | DTAG | 0.45-4.80 |  | Parks et al. 2012 |
| Cape Cod Bay | Cape Cod Bay | 3 | TDR | 0.07-0.91 |  | Baumgartner & Mate (2003); Baumgartner & Mate (2005); Baumgartner et al. (2011); Baumbartner et al. (2017). |
| Northeast | Northeast | 109 | TDR | 1.05-7.03 | 5M; 7F  2C, 5J; 40A | Baumgartner & Mate (2003); Baumgartner & Mate (2005); Baumgartner et al. (2011); Baumbartner et al. (2017). |
| Mid-Atlantic | Mid-Atlantic | 3 | SPLASH (n=2)  CATS (n=1) | 3.50-10.43 | 2C; 1A | Daniel Engelhaupt, HDR |
| Southeast | Southeast | 14 | DTAG | 0.88-23.04 | 2M; 1 F  3J; 11A | Dombroski et al. (2021) |

**Table S2: Number of tags by region and month**

| **Location** | **Month** | **n** |
| --- | --- | --- |
| Northeast | May | 50 |
| Northeast | Jun | 7 |
| Northeast | Jul | 8 |
| Northeast | Aug | 40 |
| Northeast | Dec | 2 |
| Cape Cod Bay | Mar | 3 |
| Cape Cod Bay | Apr | 16 |
| Mid-Atlantic | Mar | 2 |
| Mid-Atlantic | Nov | 1 |
| Southeast | Jan | 6 |
| Southeast | Feb | 7 |
| Southeast | Mar | 1 |

**S2. Sensitivity analysis**

**S2.1 Methods: Sensitivity analysis**

In order to better understand the influence particular parameters in the model have on overall mortality estimates, we conducted sensitivity analyses using a subset of the vessel traffic data. We used data from January and July 2022 as example months to conduct a sensitivity analysis for the following model parameters: probability at strike depth, probability of avoid, whale descent rate, whale reaction distance, vessel draft, vessel speed and whale swim speed (which impacts the encounter rate, i.e., $\lambda_{e}t$ (Figure 1)). In this analysis, we used the one-at-a-time method, where we systematically varied each model parameter while holding all other variables constant and calculated the proportional change in the total number of whale mortalities. For whale swimming speed, whale descent rate, and whale reaction distance, we used the lowest and highest 10% of realistic values (Table S4), and for the probability of time at the surface we used the lowest and highest 10% of observed values from depth-recording tags (Table S3, Table S4). For vessel draft, we calculated the model with a draft of 1.5, 3, and 5 for Small/Medium vessels and 7.5, 10, and 15 for OGVs (Table S4). We then compared the results of the model with avoidance and the model without avoidance to better understand the bounds of vessel strike risk surrounding this particular model parameter.

**S2.2 Results: Sensitivity analysis**

A comparison of two scenarios, one in which all whales attempted to avoid oncoming vessels, the other in which none of the whales attempted to avoid oncoming vessels, revealed a significant difference in estimated mortality rates, under both the real-world and slow-all scenarios. When avoidance was set equal to 0 (i.e., no whales attempt to avoid), estimated average mortality rate under the real-world scenario was over 45 individuals per year for the OGV size class and over 25 individuals per year for both the Large and Small/Medium size classes (Figure S1). Thus, mortality rates nearly doubled for each size class when the model excluded active whale avoidance (Figure S1). Spatially, areas of high risk were similar between scenarios in which avoidance was included in the model (Figure 7) and excluded from the model (Figure S2). Similar mortality rates were estimated for the slow-all simulation. Spatially, the primary difference between the two scenarios is that areas of high risk were more expansive when avoidance was excluded from the model, particularly for the Large-sized vessels within the Mid-Atlantic (Figure S2c, d), as well as the Small/Medium-sized vessels in SNE (Figure S2e, f).

The sensitivity analysis for probability at strike depth, probability of avoidance, whale descent rate, whale reaction distance, vessel draft, vessel speed and whale swim speed indicated that in addition to probability of whale avoidance, vessel draft, and whale reaction distance had the most significant impact on total whale mortality (Figure S3, Table S3). When the upper bound of whale reaction distance was used in the model (i.e., 1081-1200 m), total mortality decreased by 12.7%; however, when the lower bound was used (i.e., 10-129 m), total mortality increased by over 90%, indicating that a whale’s ability to detect and react to an oncoming vessel has a significant effect on probability of mortality. Vessel draft also has an impact on total mortality. When a vessel draft of 3 and 10 meters was used for vessels less than 350 feet and greater than 350, respectively, total mortality decreased by 38.3% (Figure S3, Table S3).

**Table S3: Depth thresholds and corresponding percent-time values used within the base model and sensitivity analysis. Depth threshold indicates the depth at which a percent number of observations above depth was calculated. Mean indicates mean percent of observations, while SD indicates standard deviation. Weighted mean indicates the mean percent of observations spent above a certain depth, weighted by tag durations. The 10th and 90th percentiles indicate the lower and upper 10% of percent of time spent above a certain depth threshold.**

| **Location** | **Depth threshold** | **Mean** | **SD** | **Weighted mean** | **10th percentile** | **90th percentile** |
| --- | --- | --- | --- | --- | --- | --- |
| Northeast U.S. | above 1.5 m | 0.20 | 0.17 | 0.16 | 0.04 | 0.43 |
|  | above 7.5 m | 0.54 | 0.23 | 0.49 | 0.27 | 0.89 |
|  | above 3 m | 0.33 | 0.18 | 0.28 | 0.12 | 0.57 |
|  | above 10 m | 0.60 | 0.25 | 0.55 | 0.29 | 0.98 |
|  | above 5 m | 0.44 | 0.20 | 0.39 | 0.21 | 0.69 |
|  | above 15 m | 0.65 | 0.27 | 0.59 | 0.31 | 1.00 |
| Cape Cod Bay | above 1.5 m | 0.65 | 0.25 | 0.66 | 0.29 | 0.85 |
|  | above 7.5 m | 0.88 | 0.20 | 0.93 | 0.66 | 1.00 |
|  | above 3 m | 0.83 | 0.23 | 0.87 | 0.54 | 1.00 |
|  | above 10 m | 0.90 | 0.20 | 0.94 | 0.67 | 1.00 |
|  | above 5 m | 0.86 | 0.21 | 0.91 | 0.63 | 1.00 |
|  | above 15 m | 0.91 | 0.19 | 0.95 | 0.69 | 1.00 |
| Mid-Atlantic U.S. | above 1.5 m | 0.55 | 0.29 | 0.54 | 0.32 | 0.79 |
|  | above 7.5 m | 0.91 | 0.08 | 0.94 | 0.84 | 0.97 |
|  | above 3 m | 0.73 | 0.17 | 0.75 | 0.59 | 0.87 |
|  | above 10 m | 0.93 | 0.07 | 0.95 | 0.87 | 0.98 |
|  | above 5 m | 0.86 | 0.09 | 0.88 | 0.79 | 0.91 |
|  | above 15 m | 0.95 | 0.06 | 0.97 | 0.90 | 0.99 |
| Southeast U.S. | above 1.5 m | 0.49 | 0.19 | 0.44 | 0.28 | 0.65 |
|  | above 7.5 m | 0.75 | 0.26 | 0.63 | 0.48 | 0.95 |
|  | above 3 m | 0.58 | 0.22 | 0.51 | 0.33 | 0.80 |
|  | above 10 m | 0.81 | 0.27 | 0.68 | 0.54 | 0.98 |
|  | above 5 m | 0.66 | 0.24 | 0.56 | 0.39 | 0.86 |
|  | above 15 m | 0.97 | 0.06 | 0.93 | 0.96 | 1.00 |


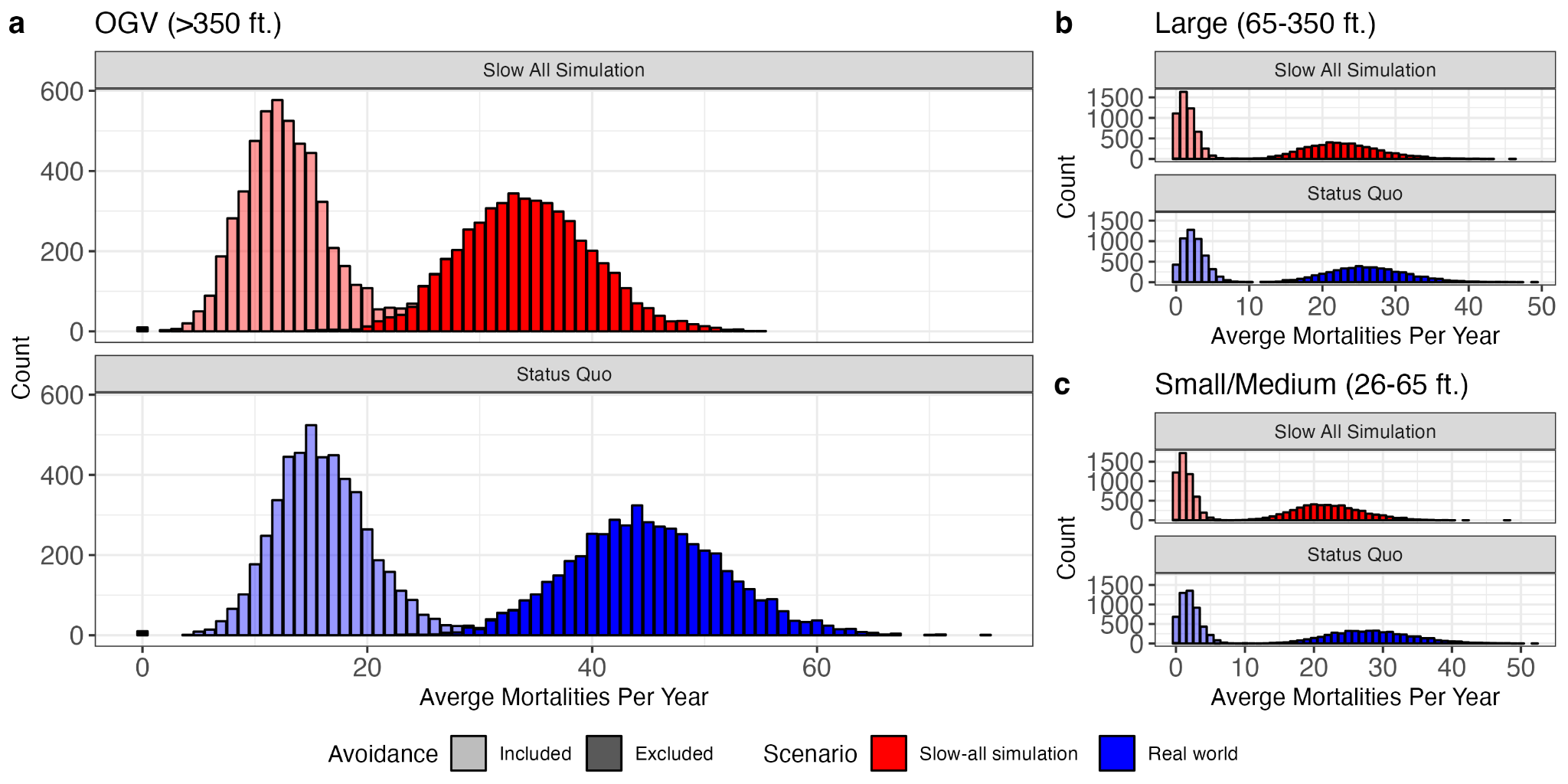


**Figure S1:** Sensitivity analysis showing a comparison of when all whales attempt to avoid an incoming vessel and no whales attempt to avoid an incoming vessel under both “Real-world” and “Slow-all” scenarios. All analyses were based on a population of 350 right whales.


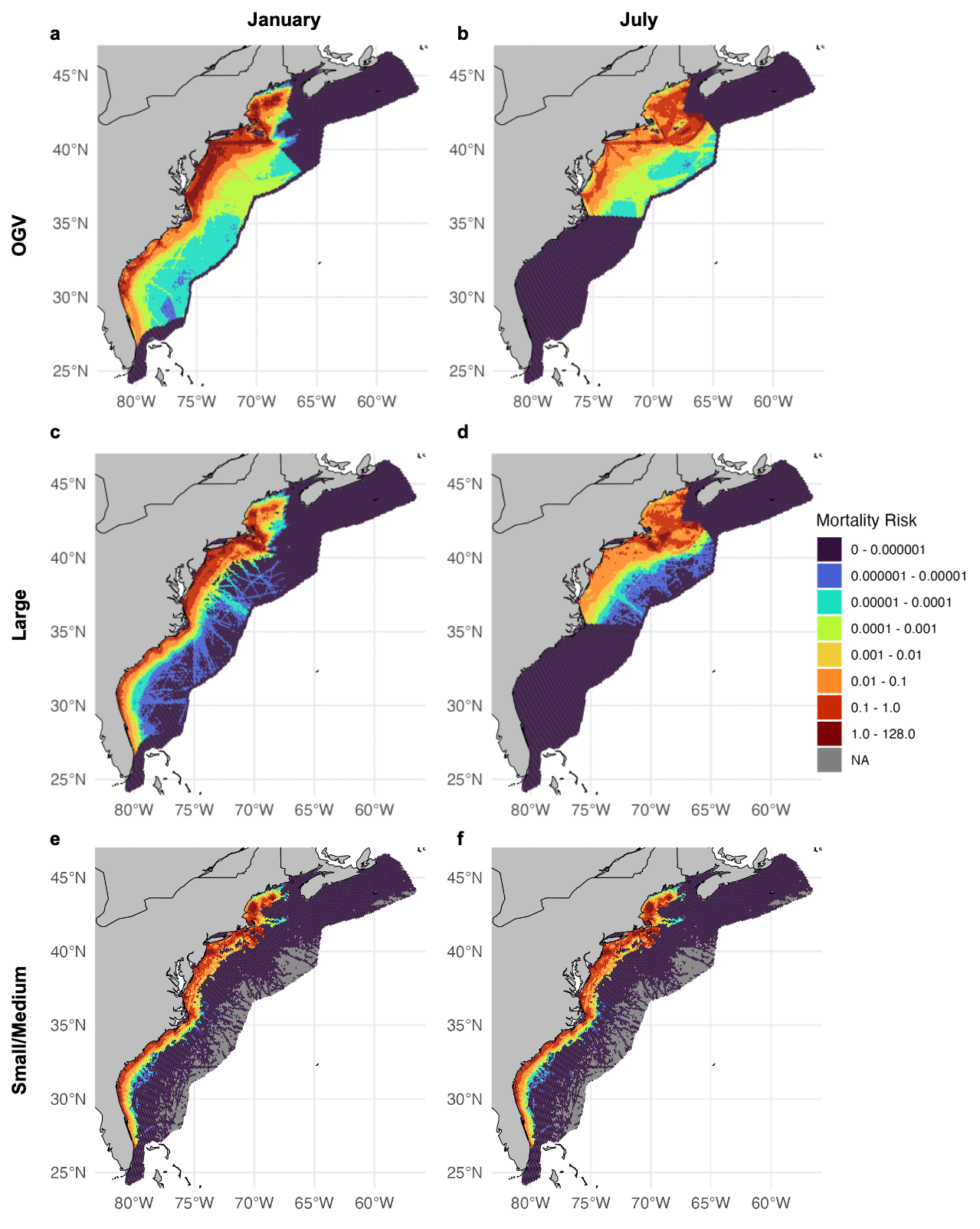


**Figure S2:** Proportion of total annual vessel strike mortality risk excluding avoidance by spatial cell for a) OGVs in January, b) OGVs in July, c) Large-sized vessels in January, d) Large-sized vessels in July, e) Small/Medium-sized vessels in January, and f) Small/Medium-sized vessels in July. All plots show the 5-year average of each spatial cell from 2017-2022, excluding 2020 within each respective month. A linear- scaling transformation was also applied to the data to better show differences in cell values among months and vessel size classes. All analyses were based on a population of 350 right whales.

**Table S4:** Sensitivity analysis iterations. This table indicates changes made to covariates in each of the sensitivity model runs. Covariate indicates the variable changed, parameter indicates the model parameter(s) impacted by this change, new values indicate the range of values included in the model run, range indicates the values relative to those used in the full model, total mean indicates the mean total number of whale mortalities resulting from the model run, S/M, L, and OGV indicate the individual mean total number of whale mortalities for each vessel size class, and change indicates if the model run resulted in a total mean number of mortalities higher or lower than the base model. Red upward arrows indicate an increase in mortalities, while a blue downward arrow indicates a decrease in mortalities.

| **Covariate** | **Parameter** | **Values used** | **Range** | **Total mean mortality (# whales)** | **S/M** | **L** | **OGV** | **Change** |
| --- | --- | --- | --- | --- | --- | --- | --- | --- |
| Base model | – | – | – | 2.857 | 0.229 | 0.237 | 2.391 | – |
| Whale descent rate | P.avoid | 0.81-0.93 m s–1 | Lower | 2.995 | 0.274 | 0.25 | 2.471 | ⇧ |
| Whale descent rate | P.avoid | 1.88-2.0 m s–1 | Upper | 2.637 | 0.225 | 0.235 | 2.177 | ⇩ |
| Whale reaction distance | P.avoid | 10-129 m | Lower | 5.438 | 0.409 | 0.312 | 4.717 | ⇧ |
| Whale reaction distance | P.avoid | 1081-1200 m | Upper | 2.493 | 0.208 | 0.272 | 2.013 | ⇩ |
| Whale depth | Prob.surface | 5 m: 0.207-0.785 | Lower | 2.366 | 0.185 | 0.217 | 1.964 | ⇩ |
|  |  | 15 m: 0.311-0.963 |  |  |  |  |  |  |
| Whale depth | Prob.surface | 5 m: 0.695-1.0 | Upper | 3.15 | 0.247 | 0.299 | 2.605 | ⇧ |
|  |  | 15 m: 0.992-1.0 |  |  |  |  |  |  |
| Vessel draft | Prob.surface, p.avoid | 26-350 ft.: 1.5 m | Lower | 0.991 | 0.072 | 0.124 | 0.795 | ⇩ |
|  |  | >350 ft.: 7.5 m |  |  |  |  |  |  |
| Vessel draft | Prob.surface, p.avoid | 26-350 ft.: 3 m | Mid | 1.762 | 0.149 | 0.158 | 1.455 | ⇩ |
|  |  | >350 ft.: 10 m |  |  |  |  |  |  |
| Probability of avoidance | p.avoid | 50% attempt | Mid | 8.015 | 2.213 | 1.792 | 4.01 | ⇧ |
| Probability of avoidance | p.avoid | 0% attempt | Lower | 12.98 | 4.171 | 3.269 | 5.54 | ⇧ |
| Whale swim speed | Encounter rate | Lower 10th  percentile: 0.00006-0.09 m/s (mean: 0.06) | Lower | 2.864 | 0.235 | 0.224 | 2.405 | ⇧ |
| Whale swim speed | Encounter rate | Upper 10th percentile: 0.76-2.32 m/s (mean: 0.96) | Upper | 2.824 | 0.218 | 0.25 | 2.356 | ⇩ |


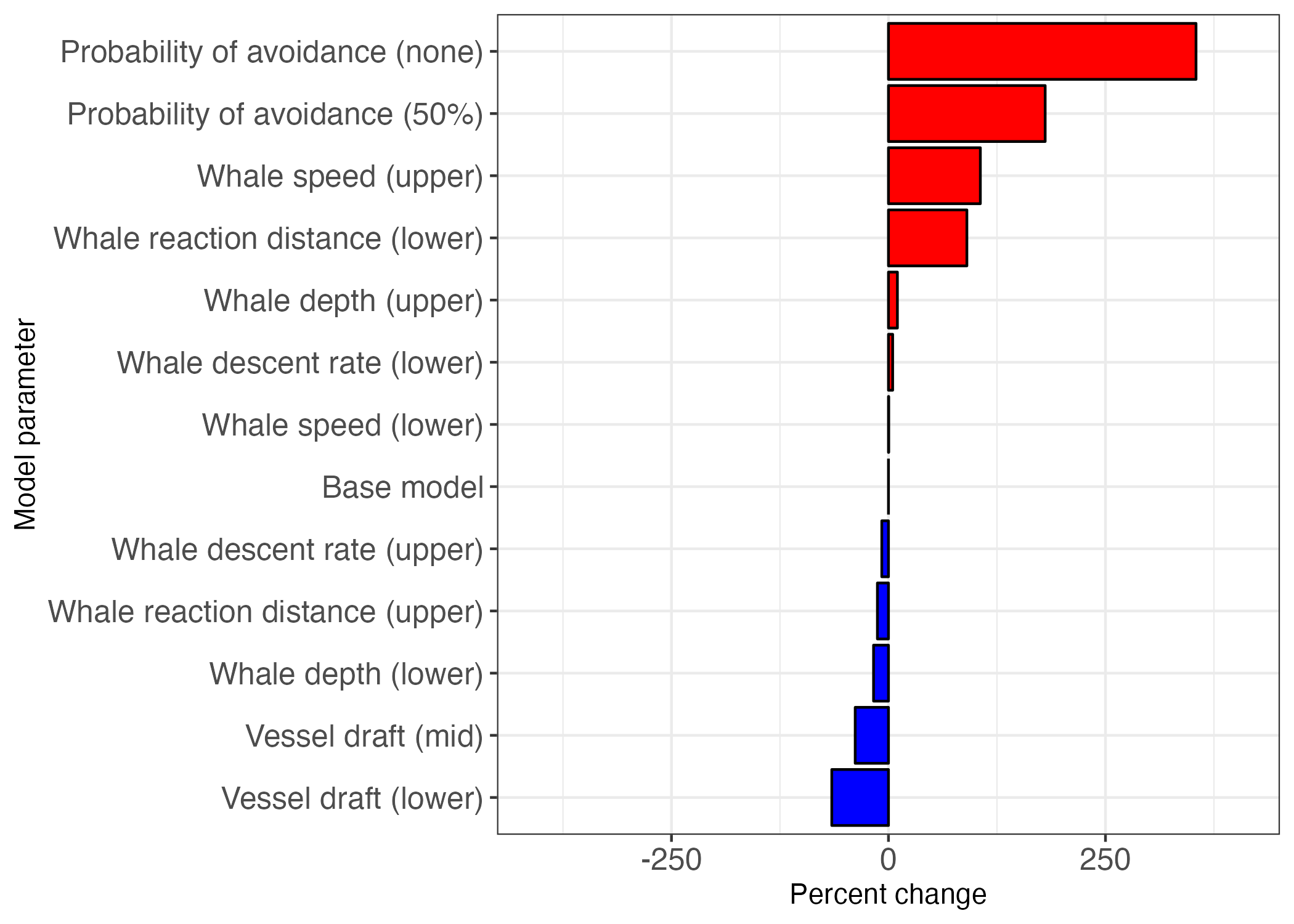


**Figure S3:** Sensitivity analysis results indicating differences in total mean mortality for two months (January and July) compared to the base model. Red bars represent increases in mean mortality while blue bars represent decreases in mortality.

**S2.3 Vessel draft**

We calculated the median and range of draft values for each of the three vessel size classes (Figure S8a) as well as the proportion of vessels in the AIS database with a draft attribute recorded and available in the database for 2022 (Figure S8b). Overall, we find a very low percentage of vessels with drafts reported in the AIS database, and a relatively high level of variability in recorded drafts. The difference between median recorded drafts and the draft used in the model (5 meters (Small/Medium and Large vessels) and 15 meters (OGVs)), is within the average height (3 meters) to length (13.5 meters) of an adult right whale. Because the encounter risk model does not take into account the orientation of a whale in the water column, and the calculation for whether or not a whale successfully avoids a vessel is based on whale height, we consider the difference between these recorded drafts and the draft we use in the model to be an appropriate estimate of vessel draft to be included within the model.


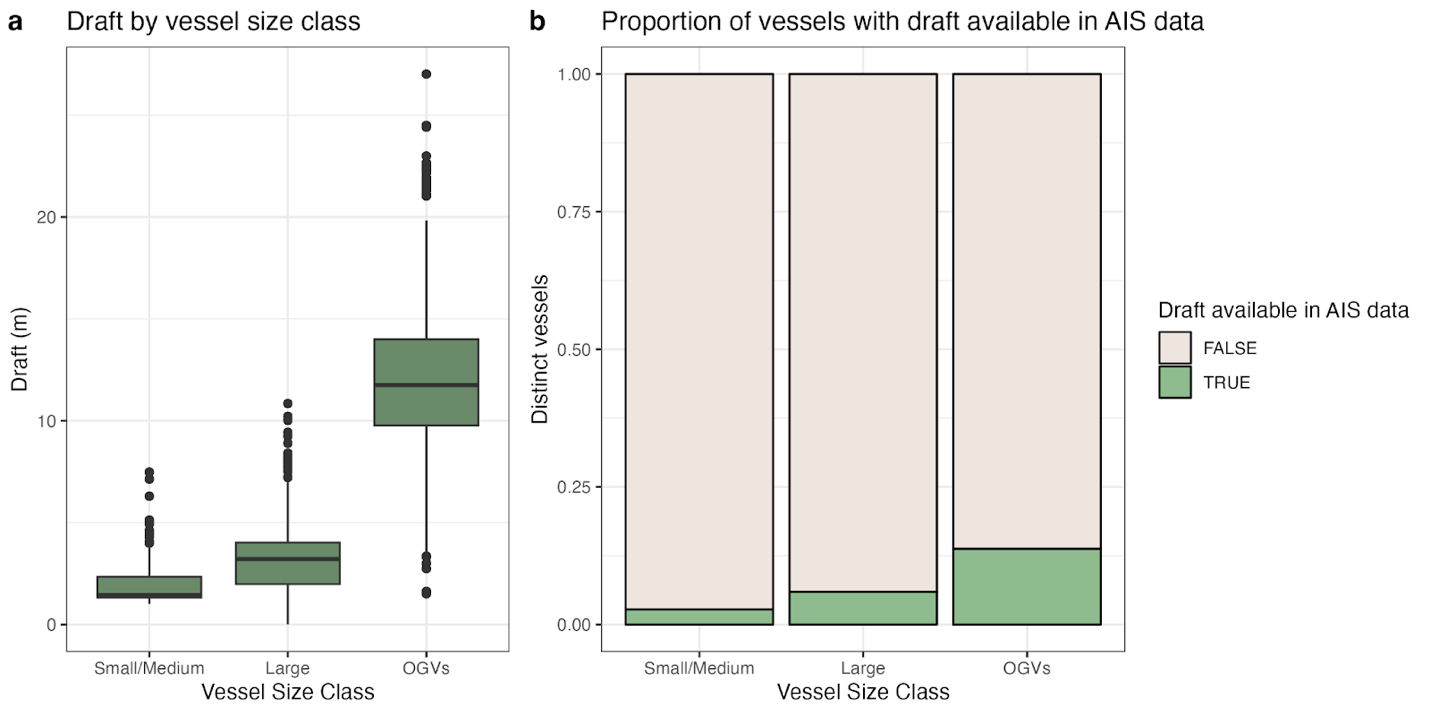


**Figure S4:** a) Boxplots showing the range of draft values for each of the three vessel size classes, including median draft in the thick black line and outlier points individually, and b) the proportion of the number of distinct vessels where draft information was available in the AIS data shown in green, versus the number of distinct vessels with draft information unavailable in tan.

### **S3. Inter-annual variability in risk model results**

**Encounter risk model results by year**

We calculated the encounter risk model for each year from 2017-2022 (excluding 2020). Levels of whale mortality are largely consistent among years within each vessel size class. Mortality under the real-world scenario increased slightly each year for the Small/Medium size class, whereas mortality varied but did not show an overall increase under the real-world scenario for OGVs and Large vessels.

**
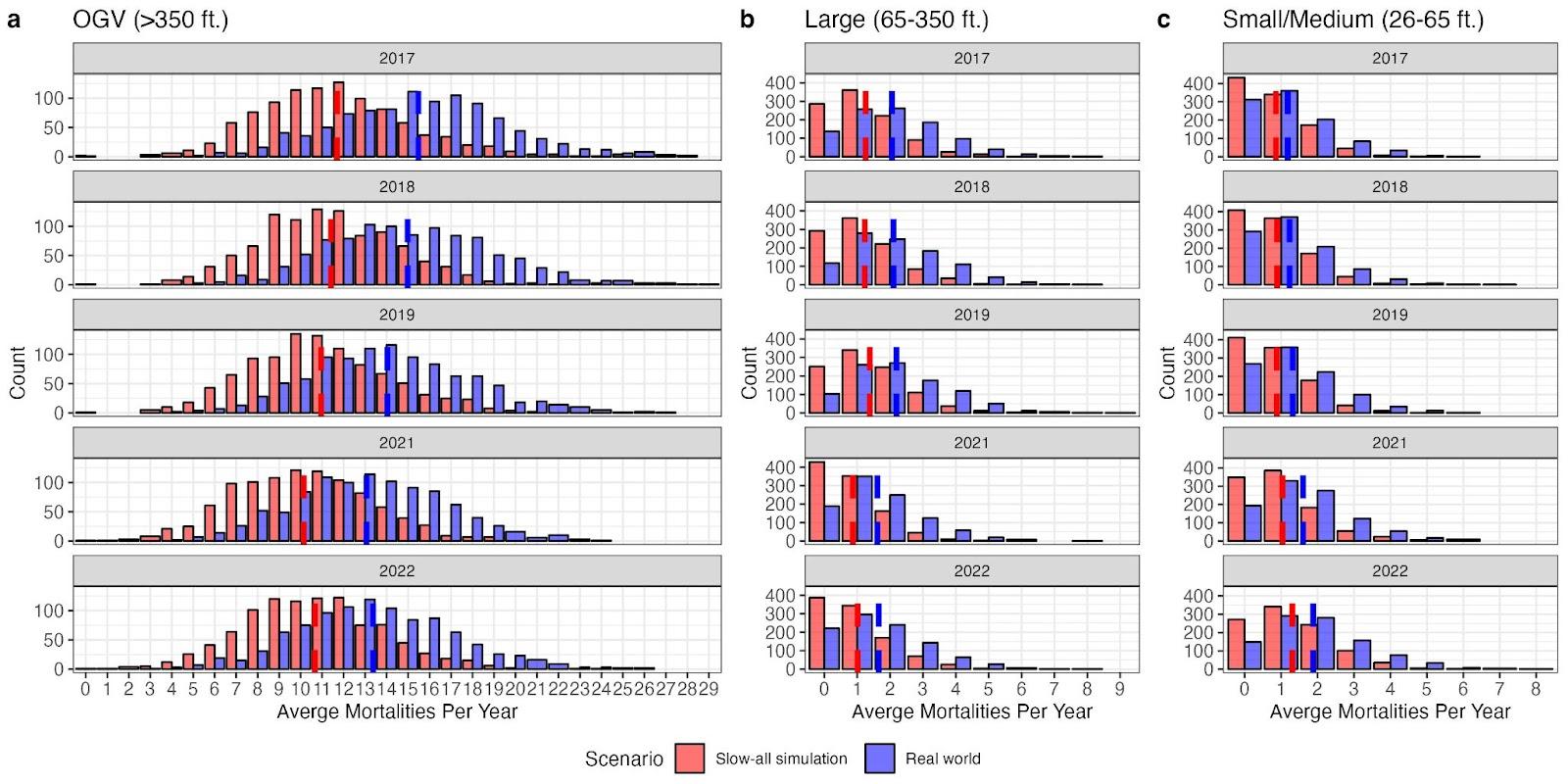
**

**Figure S5:** a) Individual bar plots of mortality rate by year for a) OGV, b) Large vessels, and c) Small/Medium vessels. All analyses were based on a population of 350 individuals. Dashed lines in each plot represent the mean number of mortalities for that year. Colors represent the corresponding scenario.

**2017**

**
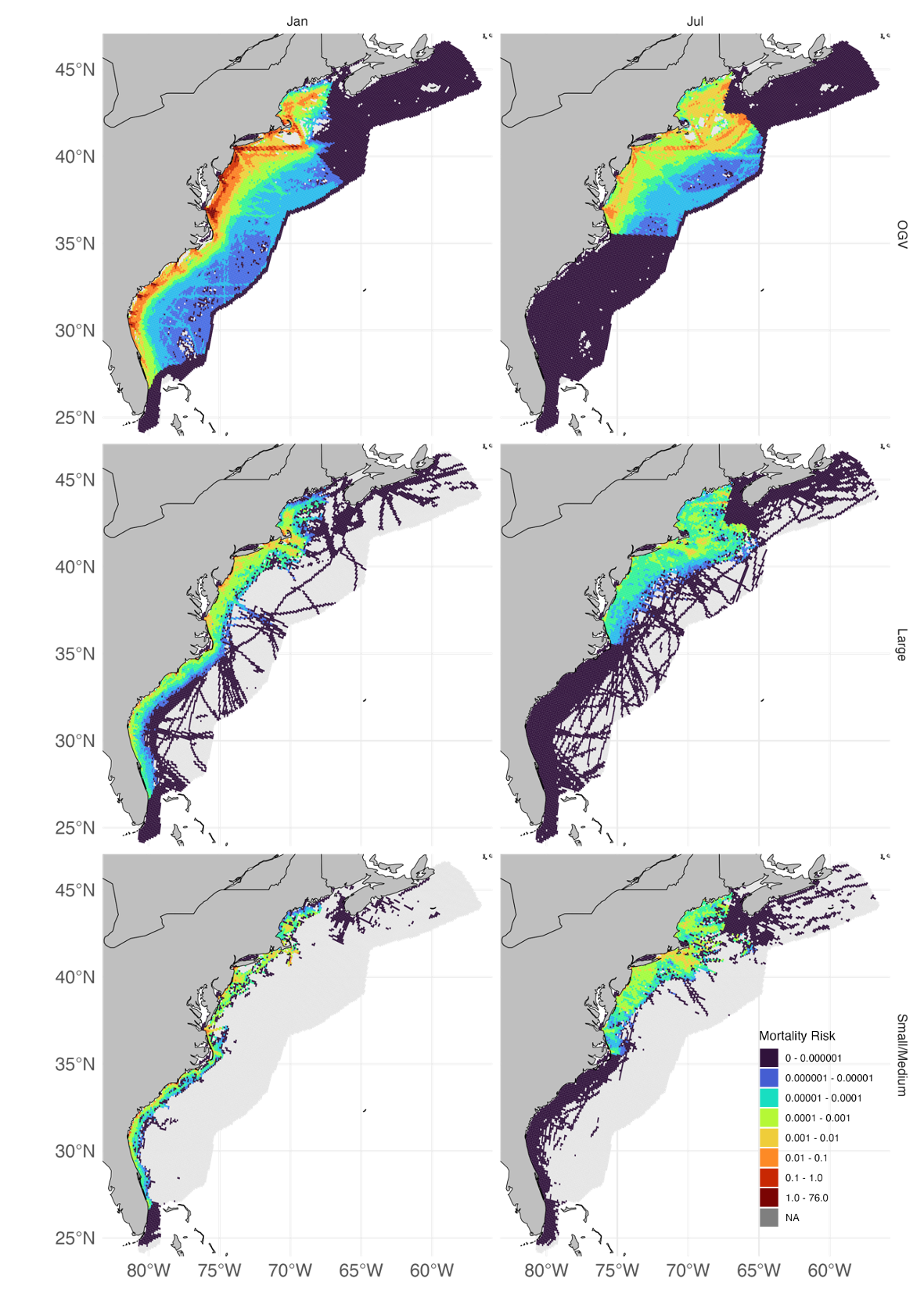
**

**Figure S6:** Proportion of total annual vessel strike mortality risk by spatial cell for a) OGVs in January 2017, b) OGVs in July 2017, c) Large-sized vessels in January 2017, d) Large-sized vessels in July 2017, e) Small/Medium-sized vessels in January 2017, and f) Small/Medium-sized vessels in July 2017. A linear- scaling transformation was also applied to the data to better show differences in cell values among months and vessel size classes. All analyses were based on a population of 350 individuals.

**2018**


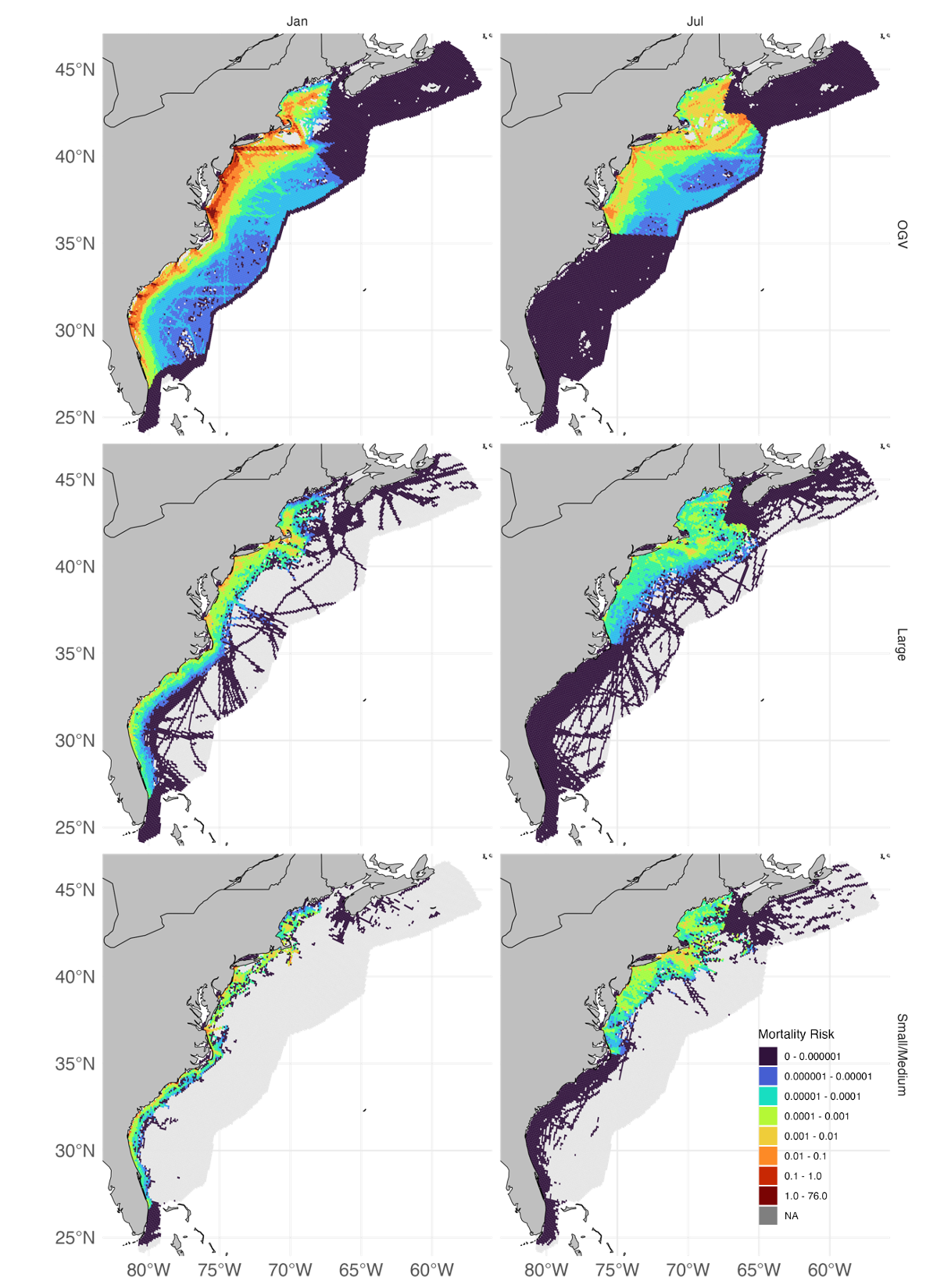


**Figure S7:** Proportion of total annual vessel strike mortality risk by spatial cell for a) OGVs in January 2018, b) OGVs in July 2018, c) Large-sized vessels in January 2018, d) Large-sized vessels in July 2018, e) Small/Medium-sized vessels in January 2018, and f) Small/Medium-sized vessels in July 2018. A linear- scaling transformation was also applied to the data to better show differences in cell values among months and vessel size classes. All analyses were based on a population of 350 individuals.

**2019**


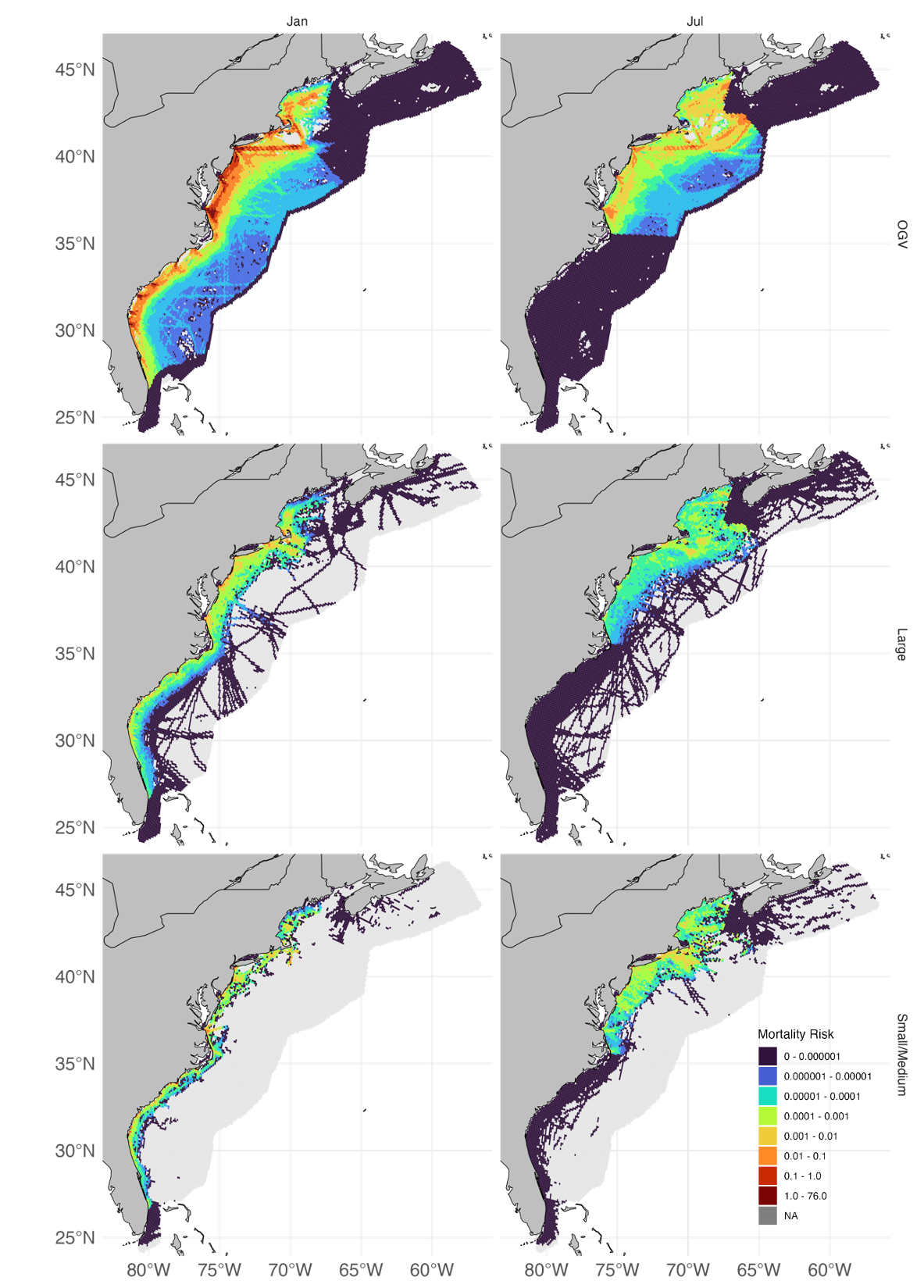


**Figure S8:** Proportion of total annual vessel strike mortality risk by spatial cell for a) OGVs in January 2019, b) OGVs in July 2019, c) Large-sized vessels in January 2019 , d) Large-sized vessels in July 2019, e) Small/Medium-sized vessels in January 2019, and f) Small/Medium-sized vessels in July 2019. A linear- scaling transformation was also applied to the data to better show differences in cell values among months and vessel size classes. All analyses were based on a population of 350 individuals.

**2021**

**
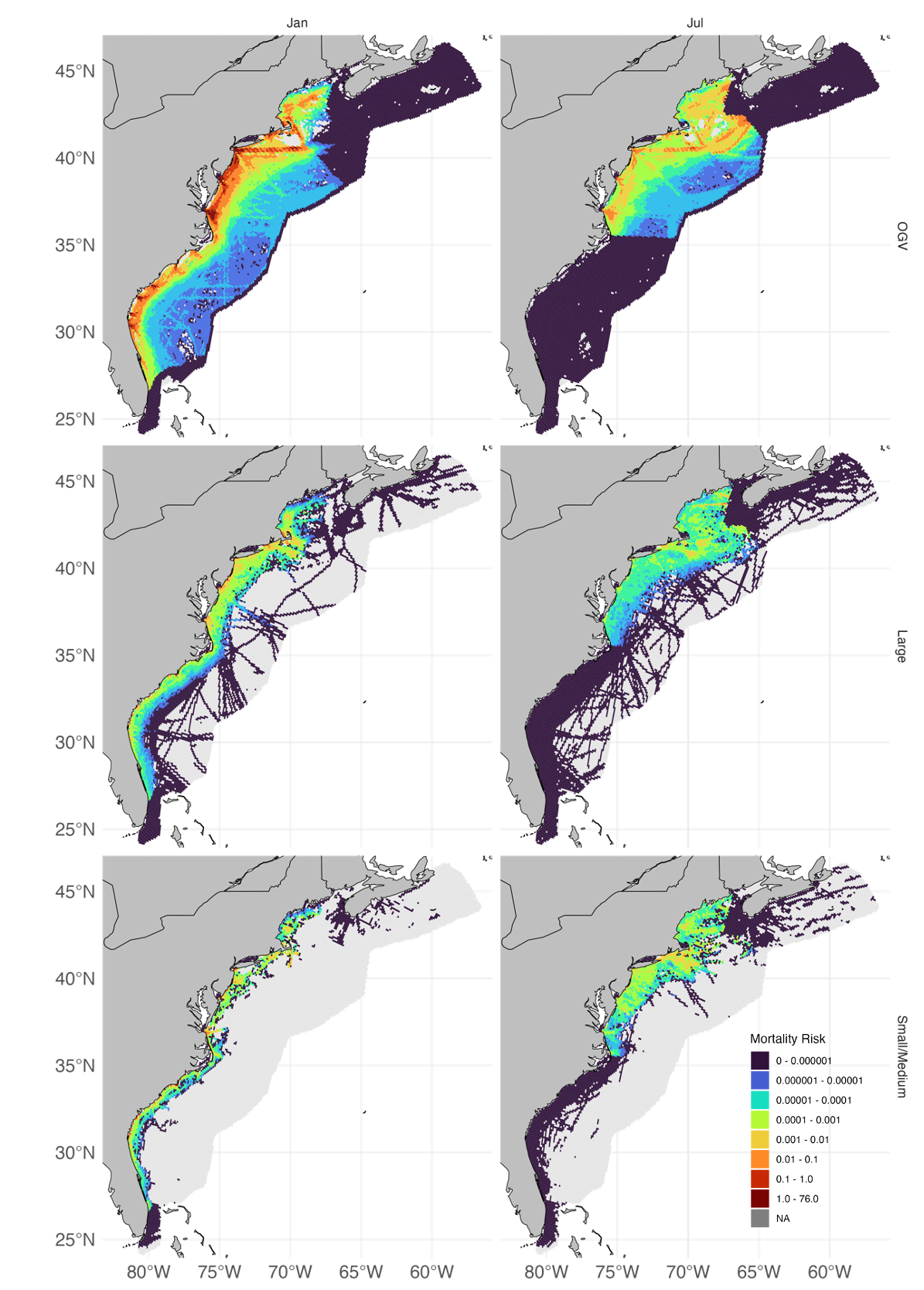
**

**Figure S9:** Proportion of total annual vessel strike mortality risk by spatial cell for a) OGVs in January 2021, b) OGVs in July 2021, c) Large-sized vessels in January 2021, d) Large-sized vessels in July 2021, e) Small/Medium-sized vessels in January 2021, and f) Small/Medium-sized vessels in July 2021. A linear- scaling transformation was also applied to the data to better show differences in cell values among months and vessel size classes. All analyses were based on a population of 350 individuals.

**2022**


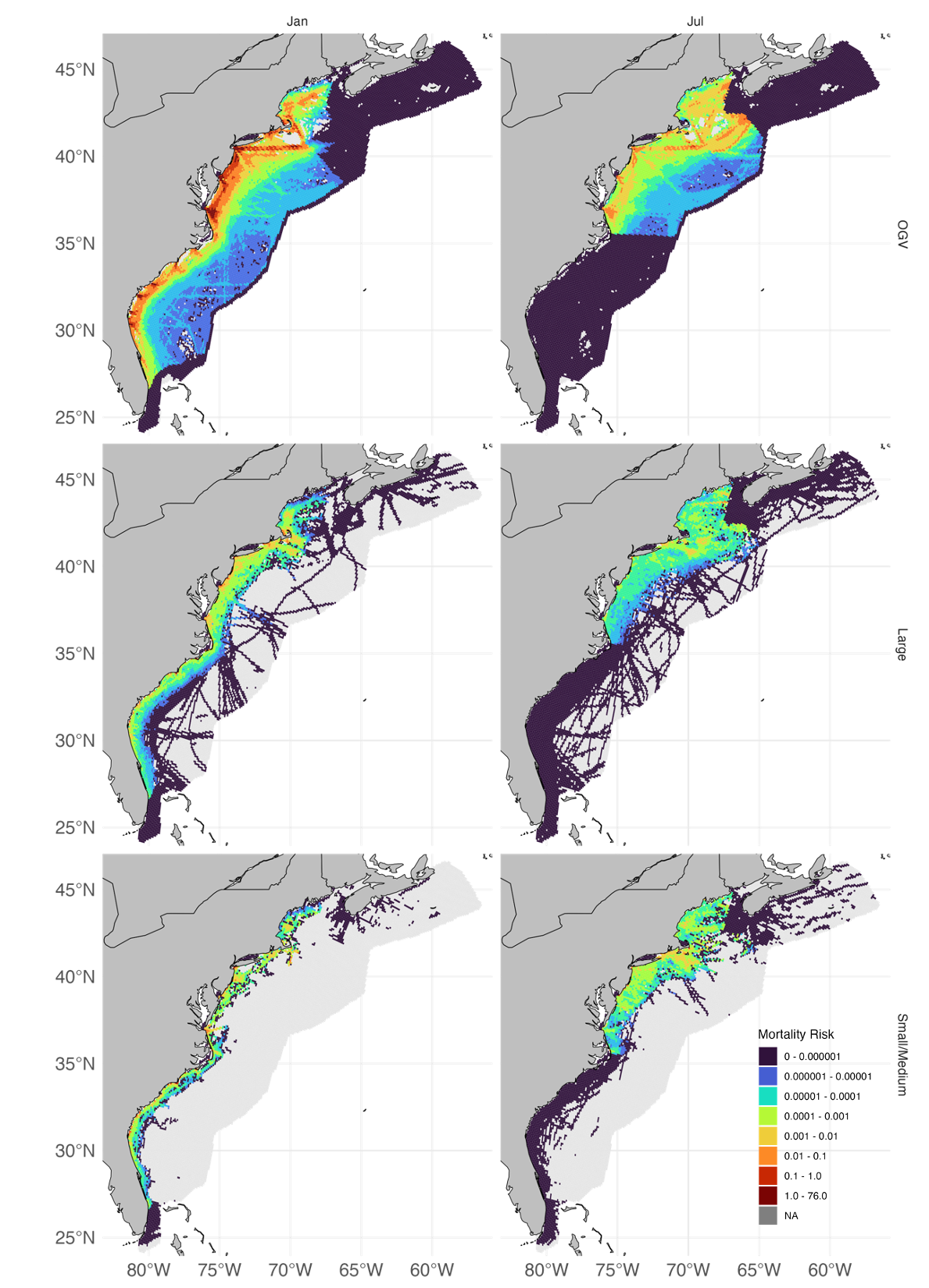


**Figure S10:** Proportion of total annual vessel strike mortality risk by spatial cell for a) OGVs in January 2022, b) OGVs in July 2022, c) Large-sized vessels in January 2022, d) Large-sized vessels in July 2022, e) Small/Medium-sized vessels in January 2022, and f) Small/Medium-sized vessels in July 2022. A linear- scaling transformation was also applied to the data to better show differences in cell values among months and vessel size classes. All analyses were based on a population of 350 individuals.

**Table S5: Table indicating implicit and explicit assumptions made within risk model, the parameter each assumption influences, and the effect of the assumption on the model output**

| **Parameter** | **Assumption** | **Effect on model output** |
| --- | --- | --- |
| Whale length | Assumes whale is average-sized adult | Does not account for calves |
| P surface | Assumes probability of whale at surface is consistent across seasons within a region | Does not account for seasonal variability in whale vertical behavior |
| Encounter rate | Assumes a vessel moves at a constant velocity through the spatial cell and in a straight path | Does not account for finer scale movements in small vessel transit behavior |
| Encounter rate | Assumes the distribution of the speed of the animal is stationary, independent of time, of the animal's position and of its orientation in the water column | Does not account for fine scale variation in whale behavior |
| Encounter rate | Assumes a maximum of one encounter between an animal and a boat during a transit | Does not account for a subsequent interaction that could occur if a whale is struck but the strike is not lethal and the whale and vessel then interact again |
| Encounter rate | Assumes that both the whale and vessel are moving randomly with respect to one another within a defined spatial area for a total amount of time, t | Does not account for vessels that may approach whales upon sighting them |
| P avoid | Assumes whale is horizontal in the water column | P avoid is based on whale height (3 m) and therefore if a whale is oriented downward or upward in the water column, the model does not account for the increase is body length available to be struck by a vessel |
| P surface; p avoid | Assumes all vessels under 350 feet have a draft of 5 m and all vessels above 350 feet have a draft of 15 m | Does not account for variability in vessel draft within vessel size classes |
| Nw | The number of right whales in the area is assumed to be distributed as a Poisson random variable each month (i.e., whales are not distributed on a finer time step than one month at a time) | Does not account for the movement of whales within the time step of a single month |
| P surface | Assumes the proportion of observations above a given depth is reflective of the probability the whale will occur above a certain depth at any given time | Does not account for finer resolution whale behavior |

**References**

Baumgartner, M. F., Cole, T. V. N., Campbell, R. G., Teegarden, G. J. & Durbin, E. G. Associations between North Atlantic right whales and their prey, Calanus finmarchicus, over diel and tidal time scales *Mar. Ecol. Prog. Ser.* **264**, 155–166 (2003).

Baumgartner, M. F. & Mate, B. R. Summertime foraging ecology of North Atlantic right whales. *Mar. Ecol. Prog. Ser* **.264**, 123–135 (2003).

Baumgartner, M. F., Lysiak, N. S. J., Schuman, C., Urban-Rich, J. & Wenzel, F. W. Diel vertical migration behavior of *Calanus finmarchicus* and its influence on right and sei whale occurrence. *Mar. Ecol. Prog. Ser.* **423**, 167–184 (2011).

Baumgartner, M. F., Wenzel, F. W., Lysiak, N. S. J. & Patrician, M. R. North Atlantic right whale foraging ecology and its role in human-caused mortality. *Mar. Ecol. Prog. Ser.* **581**, 165–181 (2017).

Dombroski, J. R. G., Parks, S. E. & Nowacek, D. P. Dive behavior of North Atlantic right whales on the calving ground in the Southeast USA: Implications for conservation. *Endang. Species Res.* **46**, 35–48 (2021).

Parks, S. E. and W. J. D. and S. K. and M. C. A. and W. D. Dangerous dining: surface foraging of North Atlantic right whales increases risk of vessel collisions. *Biol. Lett.* **8**, 57–60 (2012).
